# Direct Contact Membrane Distillation: A Critical Review of Transmembrane Heat and Mass Transfer Models

**DOI:** 10.3390/membranes16020064

**Published:** 2026-02-02

**Authors:** Nunzio Cancilla, Andrea Cipollina, Luigi Gurreri, Michele Ciofalo

**Affiliations:** 1 Dipartimento di Ingegneria, Università degli Studi di Palermo, Viale delle Scienze Ed. 6, 90128 Palermo, Italy; nunzio.cancilla@unipa.it (N.C.); michele.ciofalo@unipa.it (M.C.); 2 Dipartimento di Ingegneria Elettrica, Elettronica e Informatica, Università di Catania, Viale Andrea Doria 6 Ed. 3, 95125 Catania, Italy; luigi.gurreri@unict.it

**Keywords:** direct contact membrane distillation, heat and mass transfer, transmembrane transport model, desalination, hollow-fiber membrane, seawater

## Abstract

The present review summarizes a vast body of literature on the subject of Membrane Distillation (MD), with a special emphasis on the existing results and correlations for the transmembrane transport of heat and mass. The issue of saltwater physical properties is also discussed in depth, whereas the advective transport of heat and salt concentration in the feed and permeate compartments is only briefly mentioned but is beyond the scope of this review. The paper does not aim to provide a complete treatment of the subject of MD, which can be found in other publications. Rather, it suggests the data and correlations most suitable for the range of operating conditions typically expected in actual units implementing Direct Contact Membrane Distillation (DCMD), including hollow fiber designs, with a view to assist model development. The focus is on MD for water desalination, although some results may apply well to other fields.

## 1. Introduction

To face the growing global concern of water scarcity, desalination has expanded rapidly, with a total installed capacity of ~35 billion m^3^/year [1,2]. However, conventional desalination technologies like reverse osmosis (RO), multi-stage flash distillation (MSF), and multi-effect distillation (MED) are energy-intensive and often costly. Each cubic meter of produced fresh water consumes on the order of 2.6–8.5 kWh and emits significant CO_2_ [2,3,4,5].

**Figure 1:**
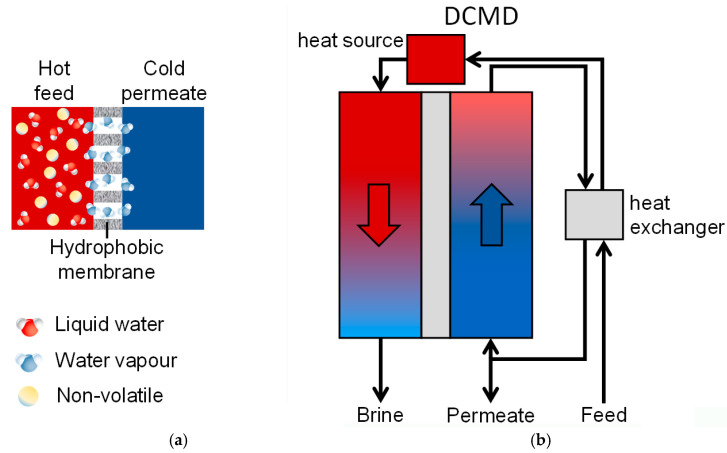
MD basic principles (**a**) and schematic of Direct Contact Membrane Distillation (DCMD) (**b**). Adapted from [17].

Membrane Distillation (MD) has emerged as a promising alternative desalination method [4,6] that could alleviate these energy and sustainability concerns [3,7]. MD is a thermally driven membrane process that operates at ambient or low pressures with a hydrophobic microporous membrane, which mediates evaporation and condensation [8,9,10,11,12,13,14]. In an MD system, a heated feed solution is brought into contact with one side of the membrane, while a cooler stream (distillate or coolant) is in contact with the opposite side (Figure 1a). The hydrophobic membrane prevents liquid penetration but allows water vapor to diffuse through its pores. A vapor–pressure gradient, maintained by the temperature difference between the hot feed and the cold permeate sides, drives water to evaporate from the feed, traverse the membrane as vapor, and condense into purified distillate on the cold side. This process yields a high-purity water product because only volatile molecules (e.g., water) pass through the membrane, leaving non-volatile solutes behind.

Notably, producing water via MD typically requires much less electrical energy (e.g., ~0.6–1.8 kWh/m^3^) since the primary energy input is thermal, and can be supplied by renewable or waste heat [3,7,9,12,15,16]. Indeed, since MD can operate at relatively low temperatures (40–80 °C), it can utilize inexpensive heat sources such as solar thermal collectors, geothermal heat, or industrial waste heat. By decoupling desalination from high electricity demands and leveraging abundant low-temperature heat, MD offers a route to reducing the carbon footprint of water production [3]. Moreover, MD offers other benefits [17]; for example, compared to RO, it requires less pre-treatment, exhibits energy consumptions which are less sensitive to the feed salinity, and has the potential for a far greater water recovery [18].

Despite its potential advantages, MD is still considered an emerging technology, with only a few pilot and full-scale implementations reported worldwide [3]. In particular, the cost-effectiveness of MD is hindered by several drawbacks [8,12,15,18,19], such as the high sensitivity to temperature polarization, risk of pore wetting, membrane fouling and scaling issues and, ultimately, uncertain energy consumption and costs, which span wide ranges [17].

MD can be configured in several modes [8,9,11,12,19,20]. In all MD configurations, evaporation at the hot side and condensation at the cold side are spatially separated by the hydrophobic membrane, but they differ in how the vapor is collected and condensed. The four classical configurations are as follows:

- Direct Contact MD (DCMD), where both the feed and permeate liquids are in direct contact with the membrane (Figure 1b);
- Air Gap MD (AGMD), where an air layer between the membrane and condensation surface serves as a barrier;
- Sweep Gas MD (SGMD), which uses a carrier gas to sweep vapor out of the membrane module to an external condenser;
- Vacuum MD (VMD), where a vacuum on the permeate side draws vapor, which then condenses externally.

DCMD is the simplest of these: in this configuration, the vapor condenses directly into the cold stream inside the module. This simplicity means that DCMD requires no specialized condensers, vacuum pumps, or sweeping gas circuits, making it easy to implement with a compact layout. Indeed, the majority of bench-scale MD studies have employed the DCMD configuration due to its straightforward design and operation [20,21]. DCMD also tends to produce higher instantaneous flux than configurations with an air gap, since the vapor path is short and the membrane is in direct contact with the cooling medium. These benefits of DCMD, together with the general advantages of MD such as low operating pressure, moderate temperature requirements, and flexibility in handling a wide range of feeds, make it attractive for diverse applications [21]. It has been successfully tested on seawater, brines from RO plants, industrial effluents, and even in the food and beverage sector for concentrating liquids like fruit juices and dairy streams.

A crucial aspect of DCMD (and MD in general) is the module design, which strongly influences heat and mass transfer efficiency. The membranes used in MD are typically available in flat-sheet or tubular formats. Flat-sheet membranes are commonly assembled into plate-and-frame or spiral-wound modules, while tubular membranes, including capillary and hollow fiber types, are integrated into shell-and-tube configurations. Plate-and-frame modules are widely adopted in laboratory-scale research due to their simple assembly, compatibility with all MD configurations, and relatively low fouling propensity. However, their packing density is limited (surface/volume ratios of 100–800 m^2^/m^3^) [9,10,22]. Spiral-wound modules offer higher compactness (surface/volume ratios of 100–1200 m^2^/m^3^) [10,22], making them more suitable for commercial use, but they tend to be more prone to fouling, and their maintenance is more problematic. Tubular, capillary, and hollow fiber modules have exhibited through the years a progressive trend towards a reduction in membrane diameter and a corresponding increase in packing density, with surface/volume ratios ranging today from 20 to 300 m^2^/m^3^ for tubular, 600 to 1200 m^2^/m^3^ for capillary, and 2000 to 5000 m^2^/m^3^ for hollow fiber modules [9,10,22]. However, this increase in packing density also correlates with a higher susceptibility to fouling, especially in hollow fiber modules, which often require appropriate pre-treatment of the feed stream.

Over the years, the MD research community has developed a variety of innovative module configurations, including hollow fiber membranes, multi-effect modules, and solar-assisted systems. The goal of new designs is to enhance the thermal energy efficiency of MD by minimizing temperature polarization, increasing mass transfer across the membrane, and improving heat recovery from condensed water vapor. A recent review by Ali et al. [23] reports advances in the design of hollow fiber and flat sheet membrane modules for MD applications, discussing current research directions in module development and highlighting potential future trends. The authors suggest that, in the coming years, the choice of module configuration will increasingly depend on the specific needs of each application. For example, in desalination and brine valorization processes, larger and more efficient spiral-wound units may become the dominant option. Conversely, hollow fiber modules featuring design modifications that reduce polarization effects (e.g., undulated fibers) may be better suited for recovering minerals and nutrients from concentrated brines and industrial effluents. Meanwhile, plate-and-frame systems equipped with flat sheet membranes are likely to be preferred in vacuum multi-effect configurations for desalination and brine concentration, as well as in AGMD setups for wastewater treatment or niche applications where ease of maintenance is a critical requirement.

The dominance of heat and mass transfer phenomena in MD performance means that rigorous modeling is indispensable for design and scale-up. Physically, DCMD involves a complex interplay of simultaneous heat and mass transport through membrane and fluid channels [8,11,12]. On the feed side, heat is consumed by evaporation at the membrane surface, cooling the adjacent liquid; on the permeate side, heat is released by condensation, warming the permeate stream. Within the membrane, heat is conducted across the polymer matrix and stagnant air in the pores, while vapor diffuses from feed to permeate driven by the vapor partial pressure gradient. Thus, mass transfer in DCMD is typically governed by a combination of molecular diffusion and Knudsen diffusion of water vapor through the pores (their relative importance depending on pore size and mean free path), whereas heat transfer encompasses convective heat transfer in the feed and permeate streams, conductive heat leak through the membrane, and the latent heat carried by the vapor [20].

These mechanisms are tightly coupled: as vapor is transported, it carries away latent heat, which in turn reduces the local temperature difference across the membrane, a critical feedback effect that can limit the driving force, referred to as temperature polarization [24,25,26]. Likewise, if the feed contains non-volatile solutes (salt, organic compounds, etc.), evaporation concentrates these solutes at the membrane interface, potentially reducing the vapor pressure. This phenomenon is known as concentration polarization and plays a minor role [27,28,29,30,31]. Because of these coupled effects, the accurate modeling of DCMD must simultaneously solve heat and mass balances in the system [20]. Typical models involve energy and mass conservation equations for the feed and permeate flow channels and within the membrane, coupled via boundary conditions at the membrane surfaces where evaporation/condensation occurs. Solving these equations can be complex, and researchers have employed a range of approaches from analytical solutions with simplifying assumptions, to numerical methods and computational fluid dynamics (CFD) [17,20,32]. Regardless of the approach, reliable models are needed to predict how operating conditions (feed temperature, flow rates, salt concentration), membrane properties (thickness, porosity, tortuosity, thermal conductivity), and module design (channel geometry, flow regime) influence the water flux and energy efficiency of DCMD. Such predictive capability is essential for process optimization and scale-up.

The present paper arises from a critical review of the literature on DCMD transmembrane models, which revealed that there is no universally adopted set of equations or standards. Different studies often employ different assumptions and correlations to describe the same underlying transport phenomena. Therefore, in the authors’ opinion, there is a need for a critical appraisal of current heat and mass transfer models for DCMD, and there is room to establish a common basis for future modeling efforts. In the present review, the authors did their best to systematically analyze recent and classical models, highlighting inconsistencies, overlaps, and gaps. This comprehensive appraisal reveals that, while numerous models exist, many can be reconciled by recognizing equivalent formulations, excluding non-justified or even non-physically based equations, and acknowledging a few errors prevalent in past studies. The paper emphasizes the need for a harmonized modeling framework based on well-validated assumptions and a consistent terminology. This paper does not aim to provide a complete treatment of the subject of MD, which can be found in other publications [8,10,20,28]. Rather, it suggests the data and correlations most suitable for the range of operating conditions typically expected in actual units implementing DCMD, including hollow fiber designs, with a view to assist model development. The focus is on MD for water desalination, although some results may apply well to other fields.

## 2. Thermophysical Properties of Salt Water

A crucial step in the prediction of MD performance is the accurate expression of the thermophysical properties of salt water (assumed here to be a simple NaCl solution) as functions of temperature and salt concentration. Several quantities are used to measure the concentration of salt in water. It is important to define them rigorously and to provide the relevant conversion formulae because different definitions are used in different correlations for physical properties and transport phenomena.

- Salinity *S*, or mass fraction, is defined as the ratio of mass of salt to mass of solution and, of course, is dimensionless and insensitive to the mass units adopted, provided they are the same for salt and the solution (although it is often measured in g/kg, i.e., as 1000·*S*, in percent, i.e., as 100·*S*, or in ppm, or parts per million, i.e., as 10^6^ *S*).
- Molar fraction *x* is defined as the ratio of moles of salt to moles of solution (water + salt) and thus, like the salinity, it is dimensionless. The quantity *x* is related to *S* as follows:
  (1) $$x=\frac{S\cdot MW_{w}}{S\cdot MW_{w}+\left(1-S\right)MW_{s}}$$
  in which *MW_w_* and *MW_s_* are the molecular weights of water and salt, expressed, as is usual, in g/mol (18 and 58.44, respectively).

- Molality *m* is defined as the ratio of moles of salt to the mass of water, expressed in mol/kg. It is related to the above-defined salinity *S* as follows:

(2) $$m=\frac{1000}{MW_{s}}\frac{S}{1-S}$$

- Molarity *M* is defined as the ratio of moles of salt to volume of solution. If expressed, as is usual, in mol/L, it is related to the above-defined salinity *S* as follows:
  (3) $$M=\frac{S\cdot \rho (S)}{MW_{s}}$$
  in which *ρ*(*S*) is the density of the solution of salinity *S*, expressed in kg/m^3^, as discussed below.

- Finally, for computational purposes, it may be preferable to express the molar concentration in moles of salt per cubic meter of solution rather than per liter, using the molar concentration *C* related to molarity *M* as follows:

(4) C=1000⋅M

For seawater Membrane Distillation, of particular importance are the case *C* = 0 (representative of the permeate, i.e., practically pure water) and the range *C* = 500–1000 mol/m^3^, representative of the feed (seawater) and the resulting retentate (brine). Lower ranges become of interest for the MD of brackish water, while higher ranges are pertinent applications of MD to desalinate hypersaline solutions (e.g., RO brine). In regard to temperature, the range of interest is typically 10 to 90 °C, the lower end corresponding to the minimum expected temperature of the permeate and the upper end to the maximum expected temperature of the feed.

An extensive and recent review of the physical properties of seawater is provided by Sharqawy et al. [33]. It includes correlations, tables and charts for density *ρ*, dynamic viscosity *µ*, specific heat *c_p_*, thermal conductivity *k*, vapor saturation pressure *p_sat_*, latent heat of vaporization *H_fg_* (present notation) and other properties. Another less recent but very extensive source of thermodynamics data, tables and correlations can be found in “Appendix A” of a textbook by El-Dessouky and Ettouney [34], who, however, omit to acknowledge the original sources of the various results presented and the expected accuracy of the various correlations. Other proposed data and correlations are scattered through the literature.

### 2.1. Density

The most accurate and recent correlation, valid to within ±0.1% in the range *t* = 0–180 °C, *S* = 0–0.16 kg/kg, is that proposed by Sharqawy et al. [33], which can be written as follows:

(5) $$\rho (t,S)=\rho_{0}(t)+\Delta \rho (t,S)$$

in which

(6) $$\rho_{0}(t)=9.999\cdot 10^{2}+2.034\cdot 10^{-2}t-6.162\cdot 10^{-3}t^{2}+2.261\cdot 10^{-5}t^{3}-4.657\cdot 10^{-8}t^{4}$$

is the density of pure water, while

(7) $$\Delta \rho (t,S)=8.020\cdot 10^{2}S-2.001S\;t+1.677\cdot 10^{-2}S\;t^{2}-3.060\cdot 10^{-5}S\;t^{3}-1.613\cdot 10^{-5}S^{2}t^{2}$$

is an additive term accounting for the influence of salinity. Similar results in the same range of temperature and salinity are provided by the more complex correlation reported by El-Dessouki and Ettouney [34], in their Equation (A.1), which, however, does not indicate its expected accuracy.

**Figure 2:**
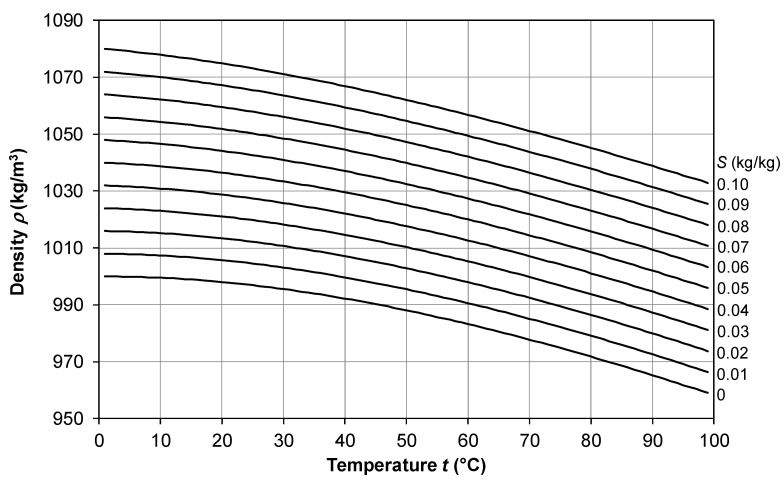
Density *ρ* of a saline solution as a function of temperature *t* for different values of the salinity *S*, based on Equations (5)–(7).

The behavior of *ρ* as a function of temperature *t* in the range 0–100 °C for different values of the salinity *S* from 0 to 0.1 kg/kg is shown in Figure 2. Note that density is affected to a similar extent by temperature and salinity; seawater (*S* = 0.03 kg/kg) at 70° has about the same density as freshwater at 10 °C.

If one wishes to express the solution density, or any other of the physical properties described in the following, as a function of the molarity *M* rather than of the salinity *S*, simply inverting Equation (3) to obtain *S* as a function of *M* yields the following:

(8) $$S=\frac{M\cdot MW_{s}}{\rho (t,S)}$$

which, however, contains the unknown density as a function of *S*. To resolve this issue, for practical purposes, *ρ* can be approximated as an explicit function of *t* and *M* as follows:

(9) $$\rho (t,M)=\rho_{0}(t)+41.6M$$

in which *ρ*_0_(*t*) is given by Equation (6). The obtained value can then be substituted for the denominator in Equation (8) to obtain in explicit form the salinity *S* as a function of *t* and *M*:

(10) $$S\approx \frac{M\cdot MW_{s}}{\rho_{0}(t)+41.6M}$$

### 2.2. Dynamic Viscosity

A correlation offering a good compromise between accuracy and range of validity is that proposed by Isdale et al. [35], declared to be valid to within ±1% in the range *t* = 10–180 °C, *S* = 0–0.15 kg/kg:

(11) $$\mu (t,S)=\mu_{0}(t)\left(1+A_{1}S+B_{1}S^{2}\right)$$

in which *µ*_0_(*t*) is the dynamic viscosity of pure water, for which the authors used the correlation proposed by Korosi and Fabuss [36]:

(12) $$\mu_{0}(t)=\exp\left(-10.7019+\frac{604.129}{139.18+t}\right)$$

while the terms *A*_1_ and *B*_1_ are the following:

(13) $$A_{1}=1.474\cdot 10^{-3}+1.5\cdot 10^{-5}t-3.927\cdot 10^{-8}t^{2}$$

(14) $$B_{1}=1.073\cdot 10^{-5}-8.5\cdot 10^{-8}t+2.23\cdot 10^{-10}t^{2}$$

The same correlation, in a slightly different formulation, is provided by El-Dessouki and Ettouney [34], in their Equation (A.3), which is declared valid in the range *t* = 10–180 °C, *S* = 0–0.13 kg/kg (however, they omit a crucial factor 10^−3^ in their Table A.3).

**Figure 3:**
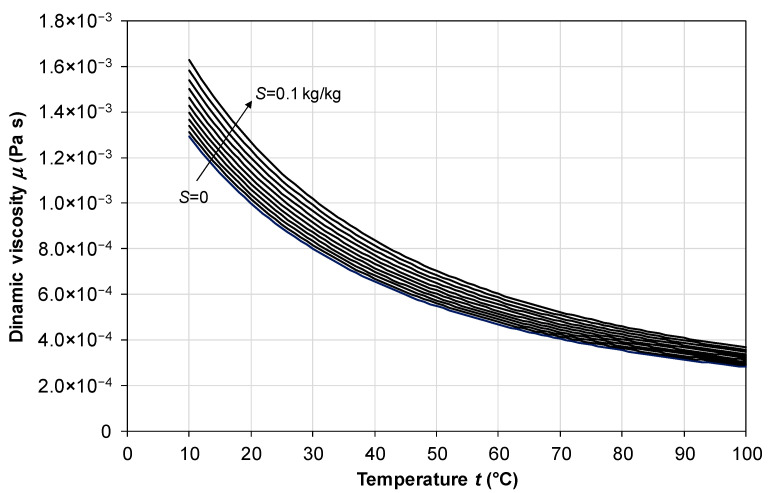
Dynamic viscosity *µ* of a saline solution as a function of temperature *t* for different values of the salinity *S*, based on Equations (11)–(14).

The behavior of *µ* as a function of temperature *t* in the range 10–100 °C for different values of the salinity *S* from 0 to 0.1 kg/kg, as predicted by Equations (11)–(14), is shown in Figure 3.

### 2.3. Specific Heat Capacity

A correlation declared to be valid to within ±0.28% in the range *t* = 0–180 °C, *S* = 0–0.18 kg/kg, is that proposed by Jamieson et al. [37]:

(15) $$c_{p}(T,S)=A_{2}+B_{2}T+C_{2}T^{2}+D_{2}T^{3}$$

in which *T* is the absolute temperature (*T* = *t* + 273.15) and the terms *A*, *B*, *C* and *D* are as follows:

(16) $$A_{2}=5.328-9.76\cdot 10^{-2}S+4.04\cdot 10^{-4}S^{2}$$

(17) $$B_{2}=-6.913\cdot 10^{-3}+7.351\cdot 10^{-4}S-3.15\cdot 10^{-6}S^{2}$$

(18) $$C_{2}=9.6\cdot 10^{-6}-1.927\cdot 10^{-6}S+8.23\cdot 10^{-9}S^{2}$$

(19) $$D_{2}=2.5\cdot 10^{-9}+1.666\cdot 10^{-9}S-7.125\cdot 10^{-12}S^{2}$$

Almost identical results, despite the different coefficients and units, are provided by the correlation reported in El-Dessouki and Ettouney [34], in their Equation (A.2), which is declared valid in the range *t* = 20–180 °C, *S* = 0.02–0.16 kg/kg.

**Figure 4:**
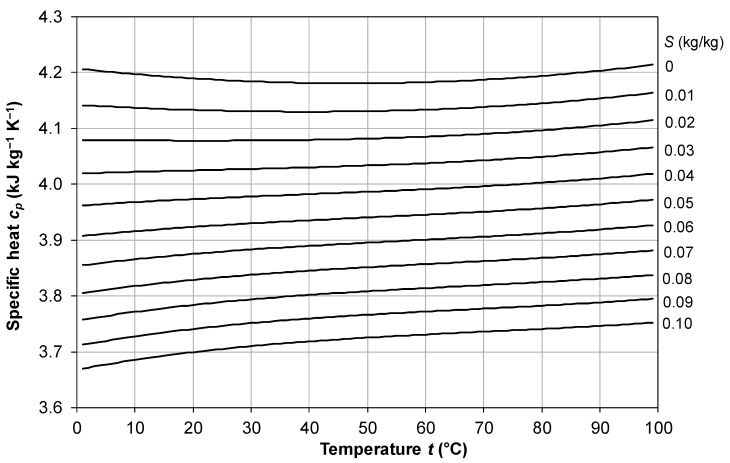
Specific heat capacity *c_p_* of a saline solution as a function of temperature *t* for different values of the salinity *S*, based on Equations (15)–(19).

The behavior of *c_p_* as a function of temperature *t* in the range 0–100 °C for different values of the salinity *S* from 0 to 0.1 kg/kg, as provided by Equations (15)–(19), is shown in Figure 4. Note the comparable influence of salinity and temperature on specific heat capacity.

### 2.4. Thermal Conductivity

Among the experimental studies presented in the literature, the most extensive (*t* = 0–180 °C, *S* = 0–0.16 kg/kg) is probably that by Jamieson and Tudhope [38], who declared their results to be approximate to within ±3% in this range of validity. Unfortunately, the correlation proposed by these authors, in their Equation (2), is obviously affected by errors and does not match the results presented by the same authors in tabular form, in their Table VI. A modified form of Jamieson and Tudhope’s correlation which matches the original experimental data was presented by El Dessouki and Ettouney [34]:

(20) $$\log_{10}(k)=\log_{10}\left(240+2\cdot 10^{-4}S\right)+0.434\left(2.3-\frac{343.5+0.037S}{T}\right){\left(1-\frac{T}{647.3+0.03S}\right)}^{\frac{1}{3}}$$

This was also reported, almost identically, by Sharqawy et al. [33] (who, however, attributed it to Jamieson and Tudhope).

**Figure 5:**
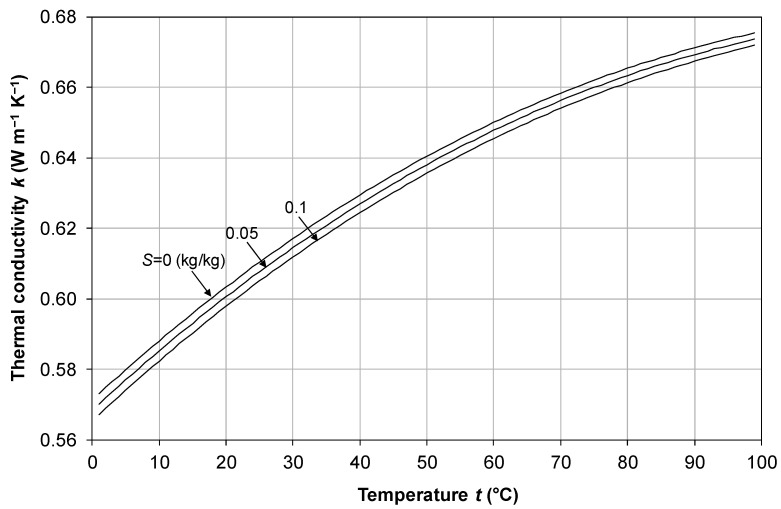
Thermal conductivity *k* of a saline solution as a function of temperature *t* for different values of the salinity *S*, based on Equation (20).

The behavior of *k* as a function of temperature *t* in the range 0–100 °C for different values of the salinity *S* from 0 to 0.1 kg/kg as predicted by Equation (20) is shown in Figure 5. Only a few curves (corresponding to *S* = 0, 0.05 and 0.1 kg/kg) are shown for clarity purposes. Note that the influence of salinity on *k* is negative and small; letting *S* increase from 0 to 0.1 kg/kg causes a decrease in *k* of only ∼1%.

Two properties of special interest, in that heat and mass transfer across a membrane are heavily influenced by them, are the vapor saturation pressure *p_sat_* and the latent heat of vaporization *H_fg_*.

### 2.5. Vapor Saturation Pressure

The vapor saturation pressure of salt water is commonly expressed by several authors, e.g., Khayet and Matsuura [28] and Alkhudhiri et al. [8], as follows:

(21) $$p_{sat}(T,\;S)=p_{sat,0}\left(T\right)a_{w}\left(S\right)$$

in which *p_sat_*_,0_ is the saturation pressure of pure water and *a_w_* is the water activity in a saline solution, expressed as a function of the salinity *S* or of any other equivalent quantity.

In its turn, *p_sat_*_,0_ is commonly expressed using Antoine’s law in one of its many different forms. In all of the following expressions, (22)–(26), the saturation pressure *p_sat_*_,0_ is in Pa and *T* is the absolute temperature in K. The following formulation of Antoine’s equation is adopted by Khayet and Matsuura [28], Yadav et al. [39], Khalifa et al. [40] and Momeni et al. [41] (in this last paper, in their Equation (12), there is a wrong “plus” sign instead of “minus” in the denominator):

(22) $$p_{sat,0}\left(T\right)=\exp\left(23.1964-\frac{3816.44}{T-46.13}\right)$$

(ranges of validity and accuracy are not provided).

Formulae possessing the same structure as Equation (22), but with slightly different numerical coefficients, are adopted by Ansari et al. [42] and Song et al. [43] and yield values of *p_sat_*_,0_ practically identical to Equation (22). Other authors, e.g., Bouguecha et al. [44], Eykens et al. [45], Kuang et al. [46], Lai and Zhang [47] and Kariman et al. [48], adopt yet another set of coefficients, which, however, yields values of *p_sat_*_,0_ ~1% larger over most of the range 0–100 °C.

Finally, a different formulation is reported by the ASHRAE Fundamentals Handbook [49]:

(23) $$p_{sat,0}\left(T\right)=\exp\left[\begin{array}{l} \frac{-5800.2206}{T}+1.3914993-4.8640239\cdot 10^{-2}T+\\ +4.1764768\cdot 10^{-5}T^{2}-1.4452093\cdot 10^{-8}\;T^{3}+6.5459673\cdot \ln\left(T\right) \end{array}\right]$$

and is claimed to be valid to within ±0.1% in the range 0–200 °C. Note that some authors, e.g., Wang et al. [50], correctly report Equation (23), while the version of the same equation reported by Sharqawy et al. [33] contains an apparently minor typo in the quadratic coefficient, but actually yields values ~5–6% too large. Equations (22) and (23) yield very similar values, the discrepancy being less than 100 Pa for *t* = 0 to 100 °C.

In regard to the dimensionless factor *a_w_* (water activity in the solution), it is generally regarded as independent of temperature. Some authors, e.g., Bouguecha et al. [44], assume *a_w_* = 1, thus neglecting altogether the influence of salinity. Several authors, e.g., Song et al. [43], Khayet and Matsuura [28], Chang et al. [51], Yadav et al. [39], Lai and Zhang [47] and Kariman et al. [48], write the activity as follows (using the present notation):

(24) $$a_{w}=x_{w}\gamma_{w}=\left(1-x\right)\gamma_{w}$$

*x_w_* = 1 − *x* being the molar fraction of water and *γ_w_* an activity coefficient. Alkhudhiri et al. [8] suggest assuming, at least as a first approximation, *γ_w_* = 1, i.e., using Raoult’s law:

(25) $$a_{w}\approx 1-x$$

Kuang et al. [46], Khalifa et al. [40] and Kariman et al. [48] adopt the following more accurate formulation:

(26) $$a_{w}=\left(1-x\right)\left(1-0.5x-10x^{2}\right)$$

As an alternative to Equation (24), Ansari et al. [42] suggest for *a_w_* the following expression:

(27) $$a_{w}=1-0.03112m-0.001482m^{2}$$

in which *m* is declared to be the “molality” but is attributed the (wrong) units of mol/L, appropriate for “molarity”.

**Figure 6:**
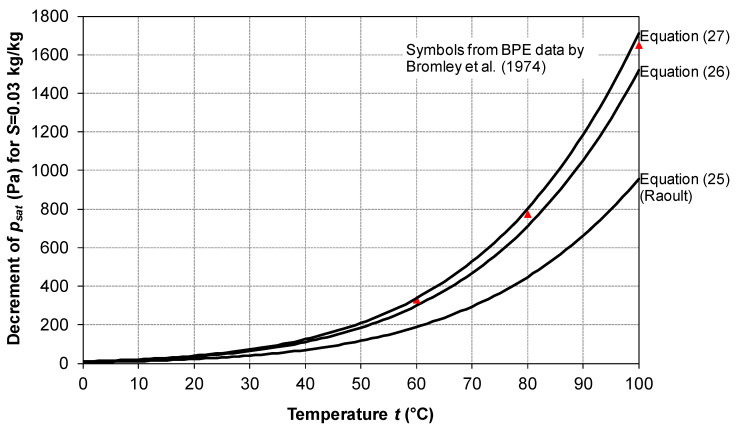
Decrement of the vapor saturation pressure *p_sat_* predicted by using alternative formulations of the water activity *a_w_* as a function of temperature *t* for a salinity *S* of 0.03 kg/kg. Data deduced from BPE measurements of Bromley et al. [52] are reported as symbols.

The saturation pressure values for salt water can also be deduced from experimental Boiling Point Elevation (BPE) data as reported by various authors, e.g., Bromley et al. [52]. A comparison between Equation (25) (Raoult’s law), Equation (26) and Equation (27) (with *m* = molality) is conducted in Figure 6 by reporting the decrement in *p_sat_* with respect to that of pure water as a function of the temperature for a salinity *S* = 0.03 kg/kg, a value typical of seawater. Values deduced from BPE measurements [52] are also reported as symbols for some temperatures. Equation (27) is that which best approximates Bromley’s data, with Equation (26) yielding only slightly lower values and the simplest Equation (25) (Raoult’s law) underpredicting the *p_sat_* decrement rather severely.

**Figure 7:**
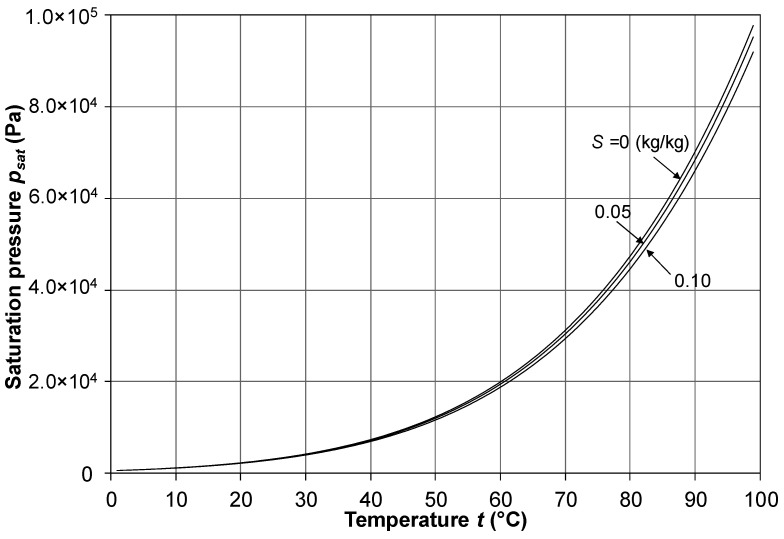
Vapor saturation pressure *p_sat_* of a saline solution as a function of temperature *t* for different values of the salinity *S*, based on Equations (21), (22) and (26).

The saturation pressure *p_sat_*, computed by using Equations (21), (22) and (26), is represented in Figure 7 as a function of temperature *t* for different values of the salinity *S* (related to the molar fraction *x* by Equation (1)). As in Figure 5, only a few values of *S* are reported for clarity purposes. The figure shows that the presence of salt up to concentrations of 0.1 kg/kg reduces only marginally the saturation pressure of a solution.

### 2.6. Latent Heat of Vaporization

According to Sharqawy et al. [33], the latent heat of vaporization *H_fg_* of a saline solution can be assumed equal to that of the liquid water mass fraction (1 − *S*) and thus can be evaluated as follows:

(28) $$H_{fg}=H_{fg,0}\left(1-S\right)$$

in which *H_fg_*_,*0*_ is the latent heat of pure water in J/kg and *S* is the salinity in kg/kg. In regard to *H_fg_*_,*0*_, Sharkawy et al. [33] propose a 4th-order polynomial fit, declared to be valid to within ±0.01% in the range *t* = 0–200 °C:

(29) $$H_{fg,0}=2.501\cdot 10^{6}-2.369\cdot 10^{3}t+2.678\cdot 10^{-1}t^{2}-8.103\cdot 10^{-3}t^{3}-2.079\cdot 10^{-5}t^{4}$$

A simpler expression which departs from Equation (29) only ~1 kJ/kg in the present range of interest (*t* = 10–90 °C) is the linear fit reported by Ansari et al. [42]:

(30) $$H_{fg,0}=2.5028\cdot 10^{6}-2.43818\cdot 10^{3}t$$

The formula reported by Khayet and Matsuura [28], Khalifa et al. [40] and other authors, i.e., *H_fg_*_,0_ = 2.0243·10^6^ + 1753.5·*T*, is obviously wrong, since it predicts *H_fg,0_* increasing with *T*.

**Figure 8:**
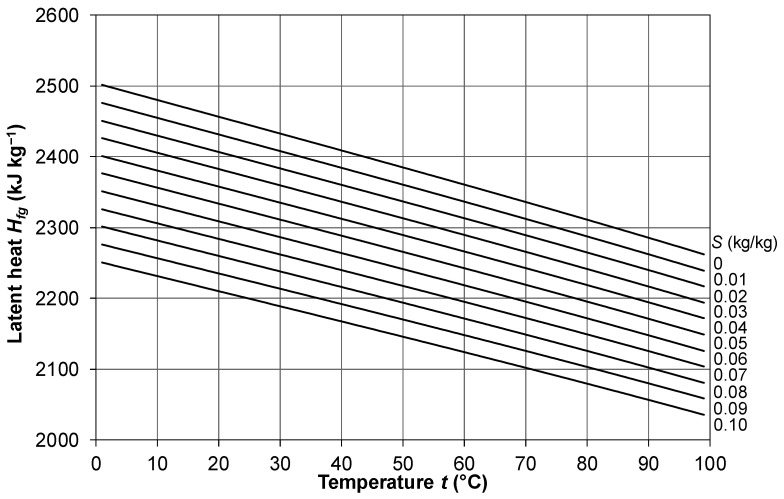
Latent heat of vaporization *H_fg_* of a saline solution as a function of temperature *t* for different values of the salinity *S*, based on Equations (28) and (29).

The behavior of the latent heat of vaporization *H_fg_* as a function of temperature *t* in the range 0–100 °C for different values of the salinity *S* from 0 to 0.1 kg/kg, as computed using Equations (28) and (29), is reported in Figure 8. Note the comparable influence of temperature and salinity on the latent heat.

## 3. Membrane Morphology and Properties

In Membrane Distillation, non-wetting, hydrophobic microporous membranes are commonly employed. Typical polymeric materials used for the fabrication of these membranes are polyvinylidene fluoride (PVDF), polytetrafluoroethylene (PTFE), polypropylene (PP) and polyethersulfone (PES). Ideally, optimal membranes adopted in MD systems should facilitate efficient (i.e., low) resistance to mass transfer while exhibiting minimal thermal conductivity to minimize heat dissipation across the membrane. Furthermore, they ought to maintain structural integrity at elevated temperatures and demonstrate robust tolerance to corrosive substances, including acids and alkalis. There are several membrane properties, such as thermal conductivity, porosity, pore size distribution and tortuosity, the mean free path of water vapor molecules within the pores and the wetting pressure, for which correlations and equations can be found in the literature to allow for their estimation. These will be presented in the following subsections.

### 3.1. Membrane Thermal Conductivity

According to various authors, such as Alkhudhiri et al. [8], Khayet and Matsuura [28], Zhang et al. [53] and Olatunji and Camacho [20], most models for the effective thermal conductivity *k_m_* of the membrane as a whole can be reconducted to either of the following expressions:

(31) $$k_{m}=\left(1-\varepsilon \right)k_{sm}+\varepsilon k_{g}$$

(32) $$\frac{1}{k_{m}}=\frac{\left(1-\varepsilon \right)}{k_{sm}}+\frac{\varepsilon }{k_{g}}$$

where *k_sm_* is the thermal conductivity of the solid matrix (polymer), *k_g_* is the thermal conductivity of filling gas (air and/or water vapor), and *ε* is the porosity of the membrane. Equation (31) (called the “parallel”, “Voigt” or “isostrain” model by analogy with the elastic modulus of composites) is preferred by Chang et al. [51], Yadav et al. [39], Schofield et al. [54], Momeni et al. [41] and several other authors, while Equation (32) (“series”, “Reuss” or “isostress” model) is preferred by Phattaranawik et al. [55].

Garcıa-Payo and Izquierdo-Gil [56] compared nine different models for the prediction of the membrane effective thermal conductivity against experimental data for two PVDF, two unsupported PTFE and two supported PTFE membranes. They concluded that the parallel model (31) largely overestimates the thermal conductivity, whereas the series model (32) slightly underestimates it. The best fit for all tested membranes, especially at large porosities (>60%), was found to be obtained by the following formula, recommended also by Hitsov et al. [32] and Ansari et al. [42]:

(33) $$k_{m}=k_{g}\frac{1+2\zeta \left(1-\varepsilon \right)}{1-\zeta \left(1-\varepsilon \right)}$$

in which the coefficient *ζ* is

(34) $$\zeta =\frac{k_{sm}-k_{g}}{k_{sm}+2k_{g}}$$

**Figure 9:**
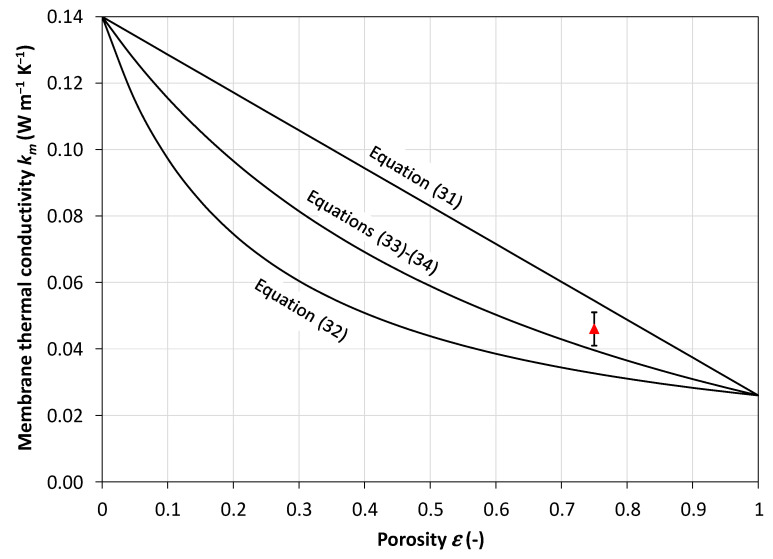
Effective thermal conductivity km of the membrane predicted by three different models (see text) for *k_sm_* = 0.14 W m^−1^ K^−1^ and *k_g_* = 0.026 W m^−1^ K^−1^. The experimental result of Schofield et al. [54] is also reported with error bars.

Figure 9 compares the values of the membrane effective thermal conductivity *k_m_* (in W m^−1^ K^−1^), predicted as a function of the porosity *ε* by using Equation (31) (parallel model), Equation (32) (series model), and Equations (33) and (34) (combined model) for *k_sm_* = 0.14 W m^−1^ K^−1^ (representative of PP at 25 °C) and *k_g_* = 0.026 W m^−1^ K^−1^ (representative of air at the same temperature). The experimental value measured by Schofield et al. [54] for dry air-filled Enka PP membranes with a thickness of 0.1 mm, porosity of 0.75, and nominal pore size of 0.1 µm (*k_m_* = 0.046 ± 0.005 W m^−1^ K^−1^) is also reported with error bars.

All three models correctly converge to *k_sm_* in the limiting case *ε* = 0 and to *k_g_* in the limiting case *ε* = 1, but the values predicted at intermediate porosities vary broadly. The experimental result of Schofield et al. [54] is overpredicted by ~13% by the parallel model, which yields *k_m_* = 0.052 W/(m K); underpredicted by ~15% by the combined model, which yields *k_m_* = 0.039 W/(m K); and grossly underpredicted (by ~30%) by the series model, which yields *k_m_* = 0.0325 W/(m K). Note that these results partially contradict the remarks by Garcıa-Payo and Izquierdo-Gil [56] mentioned above. According to Schofield et al. [54], it would be expected that most commercial microfiltration membranes would have a thermal conductivity between 0.04 and 0.06 W/(m K), increasing with decreasing porosity.

The application of any of the above formulae requires a knowledge of the thermal conductivity of the pore-filling gas and of the polymeric matrix, both preferably evaluated at the membrane mean temperature 〈*t_m_*〉 = (*t_f,m_* + *t_p,m_*)/2 (where *t_f,m_* and *t_p_*_,*m*_ are the feed–membrane and membrane–permeate interface temperatures, respectively). In regard to the gas phase conductivity *k_g_*, the membrane pores can be assumed to be filled by a mixture of air and water vapor. For air, accurate thermal conductivity values are reported by Stephan and Laesecke [57]. In the temperature range of interest (*t* = 10–90 °C) and at a pressure *p* of 1 bar, they can be approximated with excellent accuracy by the following quadratic formula:

(35) $$k_{air}=-4.107\cdot 10^{-8}t^{2}+7.631\cdot 10^{-5}t+2.417\cdot 10^{-2}\;\;\;$$

Practically identical results are provided in the range of interest by the simpler (linear) formula adopted by Hitsov et al. [32] and Ansari et al. [42]:

(36) $$k_{air}=\;7.77\cdot 10^{-5}t+\;2.394\cdot 10^{-2}\;\;\;$$

Alkhudhiri et al. [8] report the following even simpler formula:

(37) $$k_{air}=1.5\cdot 10^{-3}\sqrt{T\;}\;\;\;$$

which, however, approximates Equations (35) and (36) only at low temperatures (~300 K) but departs from them at higher temperatures and thus is not recommended. Also, the following formula is reported by Olatunji and Camacho [20]:

(38) $$k_{air}=1.36\cdot 10^{-3}+3.885\cdot 10^{-5}T+1.66\cdot 10^{-3}\sqrt{T\;}\;\;\;$$

This is unacceptable, since it predicts excessively high values of *k_air_* at all temperatures.

The influence of pressure is very small, and thus the above values, Equation (35) or Equation (36), can be used with confidence for the air filling the membrane pores with its partial pressure (possibly slightly less than atmospheric pressure).

For water vapor, properties (including thermal conductivity *k_vap_*) should be evaluated along the saturation line rather than at a constant pressure. The NIST data [58] can be used, which can be fitted in the range of interest using the following simple quadratic formula:

(39) $$k_{vap}=\;1.6185\cdot 10^{-7}t^{2}\;+\;6.1488\cdot 10^{-5}t\;+\;1.6779\cdot 10^{-2}$$

**Figure 10:**
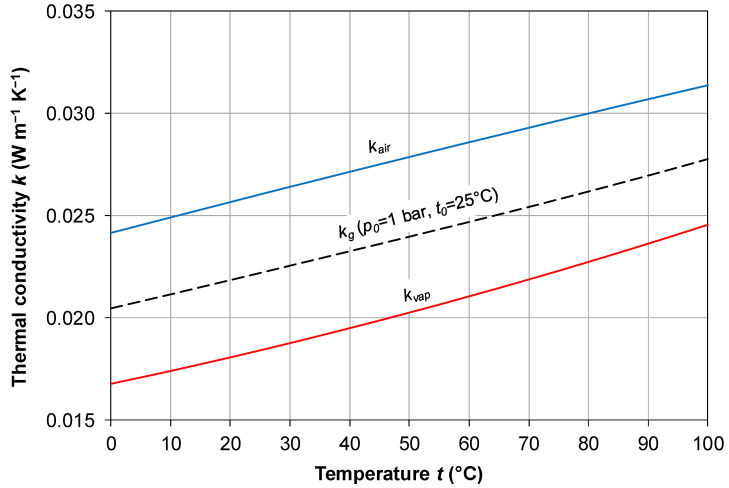
Solid lines: thermal conductivities in W/(mK) of air at *p* = 1 bar (*k_air_*) and water vapor along its saturation line (*k_vap_*), computed by using Equations (35) and (39), respectively, as functions of temperature *t* in °C. Broken line: thermal conductivity of the gas mixture filling the membrane pores according to Equations (40) and (41) for *t*_0_ = 25 °C, *p*_0_ = 1 bar.

The thermal conductivities of air at *p* = 1 bar (*k_air_*) and water vapor along its saturation line (*k_vap_*) are reported as solid lines in Figure 10 as functions of temperature *t* in the range 0–100 °C, computed by using Equations (35) and (39), respectively.

In regard to the thermal conductivity of the mixture of air–water vapor (pore filling gas), calculating it as a simple weighted average of *k_air_* and *k_vap_*, using the molar fractions (or relative partial pressures) as weights, can be inaccurate. According to Udoetok [59], for binary mixtures, better results are obtained using the following formula (present notation):

(40) $$k_{g}=\;0.5\cdot \frac{k_{air}k_{vap}}{x_{air}k_{air}+x_{vap}k_{vap}}\;+\;0.5\cdot \left(x_{air}k_{air}+x_{vap}k_{vap}\right)$$

which expresses the conductivity *k_g_* of the gas mixture as an average between the values that would be obtained by assuming the two fractions as thermal conductances in series or in parallel. The molar fractions *x* of air and water vapor in the membrane pores can be estimated under the assumptions that, before operation, the pores are filled by air only at an absolute temperature *T*_0_ and pressure *p*_0_ and that neither the pore volume nor their air content change during operation. Therefore, at a mean membrane absolute temperature *T*, the partial pressure of air is *p_air_* = *p*_0_*T*/*T*_0_, while the partial pressure of water vapor can be identified with *p_sat_*(*T*). The molar fractions to be used in Equation (40) are then given by the following:

(41) $$x_{vap}=\;\frac{p_{sat}(T)}{p_{sat}(T)+p_{0}T/T_{0}};\;\;\;\;\;\;\;\;\;\;\;x_{air}=\;\frac{p_{0}T/T_{0}}{p_{sat}(T)+p_{0}T/T_{0}}$$

For example, assuming *p*_0_ = 1 bar (10^5^ Pa) and *t*_0_ = 25 °C (*T*_0_ = 298.15 K), and using Equations (35) and (39) for *k_air_* and *k_vap_*, Equations (40) and (41) yield for *k_g_* the behavior shown in Figure 10 as a broken line. As expected, *k_g_* is an intermediate between *k_air_* and *k_vap_*; it can be verified that moderate changes in *p*_0_ and *t*_0_ affect the computed values of *k_g_* only negligibly.

In regard to the conductivity of the solid polymeric matrix, *k_sm_* (the subscript “*sm*” is for “solid matrix”), according to Alkhudhiri et al. [8] and Khayet and Matsuura [28], the typical values of *k_sm_* at 23 °C (~296 K) are in the range 0.17–0.19 W m^−1^ K^−1^ for PVDF, 0.25–0.27 W m^−1^ K^−1^ for PTFE and 0.11–0.16 W m^−1^ K^−1^ for PP. In addition, Khayet and Matsuura [28] report thermal conductivities at 348 K (~75 °C) of 0.21 W m^−1^ K^−1^ for PVDF, 0.29 W m^−1^ K^−1^ for PTFE and 0.2 W m^−1^ K^−1^ for PP.

Hitsov et al. [32], based mainly on data from Phattaranawik et al. [55], account for the temperature variation in *k_sm_* through the following linear fit:

(42) $$k_{sm}=\alpha {\cdot 10}^{-4}T+\;\beta {\cdot 10}^{-2}$$

where *α* and *β* are reported in Table 1 for different membrane materials. The same correlations are used by Ansari et al. [42] and Olatunji and Camacho [20].

**Figure 11:**
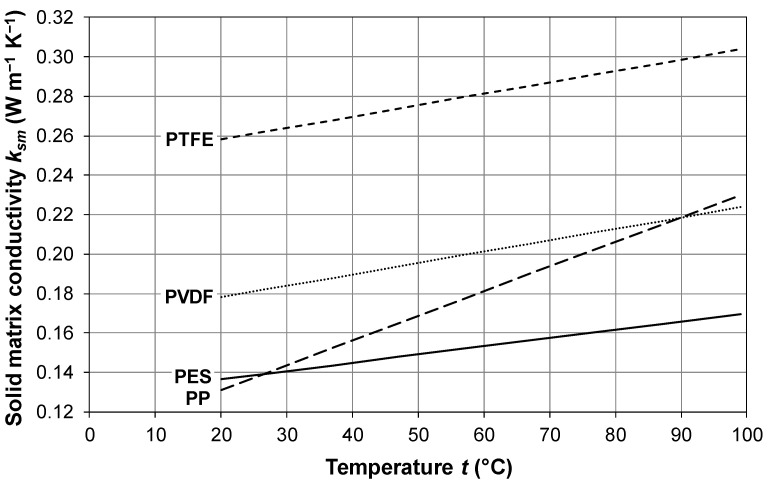
Thermal conductivity of the solid matrix for polymeric materials commonly adopted in membranes for MD, *k_sm_*, as a function of temperature *t* in °C. Based on literature correlation in Equation (42).

The values of *k_sm_* for the same materials are represented as functions of temperature *t* in the range 20–100 °C in Figure 11.

For PTFE, Alkhudhiri et al. [8] report the following correlation:

(43) $$k_{sm}=4.86\cdot 10^{-4}t+0.253$$

which correlates *k_sm_* with the temperature *t* in Celsius rather than with the absolute temperature *T*. Equation (43) yields values similar to Equation (42), written for PTFE.

As a word of caution, it must be kept in mind that the conductivity data for polypropylene are highly scattered and depend on various factors such as crystallinity, the presence of additives and the kind of treatment performed. The data reported above lie in the lower range of experimental values from the literature.

### 3.2. Pore Size and Its Distribution

El-Bourawi et al. [60] and Lawson and Lloyd [61] report that, in MD systems, the pore size usually ranges between 100 nm and 1 μm. In general, the larger the pore size, the higher the permeate flux; on the other hand, the smaller the pore size, the lower the liquid penetration. For every feed solution and operating condition, the optimal pore size should be established. Moreover, the mass transfer mechanism varies with the pore size, as will be discussed in Section 4.

The pore diameter in an MD membrane is not uniform but follows a statistical distribution. A uniform pore size distribution is typically desirable in MD systems to enhance overall efficiency. A narrow distribution can also be advantageous, as it helps prevent the wetting of the largest pores or a subset of pores, which would otherwise reduce the effective membrane surface area and diminish membrane performance. Uniform pores lead to steadier fluxes and more reliable operation, particularly during extended use [28], whereas membranes with a large and non-uniform pore distribution are susceptible to wetting, which compromises the salt rejection efficiency [62].

Phattaranawik and Jiraratananon [63] evaluated the pore size distribution in PVDF and PTFE membranes by using scanning electron microscopy (Hitachi model S-900, Tokyo, Japan) and image processing software (AnalySIS provided by Soft Imaging System). They found that pore size is a broadly scattered variable, and even the mean pore diameter does not always comply with the nominal data provided by the manufacturer. For example, in PTFE Sartorious membranes with a nominal pore diameter of 0.2 μm, they measured an average pore diameter of 0.253 μm, with more than 10% of the pores above a diameter of 0.4 μm and ~5% below 0.1 μm. They suggested the log-normal law:

(44) $$n\left(d_{p}\right)=\frac{1}{SD_{\log}d_{p}\sqrt{2\pi }}\exp\left\{-\frac{1}{2}{\left[\frac{\ln\left(d_{p}/\langle d_{p}\rangle \right)}{SD_{\log}}\right]}^{2}\right\}$$

where *d_p_* and 〈*d_p_*〉 are the generic and mean pore diameter, respectively, and *SD*_log_ is the standard deviation of the dimensionless variable *d_p_*/〈*d_p_*〉. The dimensionless quantity exp(*SD*_log_) is called the geometric standard deviation of the distribution.

More recently, Fan et al. [64] measured the pore size distribution in PVDF membranes using a gas–liquid displacement method. A membrane sample was first soaked in n-butanol, and then the wetting liquid was displaced by ultra-high purity N_2_ gas with a stepwise increase of pressure, so that the largest pores were voided first while increasingly smaller pores required increasingly higher pressures, as dictated by the Young–Laplace equation. As remarked by Dong et al. [65], most models of transmembrane mass transfer make use of a mean pore size and could incur significant error due to the distribution of the pore size. In particular, Khayet et al. [66], using their theoretical model, found that the predicted permeance increases as the geometric standard deviation of the pore size distribution increases. The increment in membrane permeance is small in commercial membranes, which generally exhibit a narrow pore size distribution, whereas larger discrepancies may arise when laboratory-made membranes with broader pore size distributions are used. Woods et al. [67] used a numerical model to predict the water vapor flux through all pore sizes and estimate the effect of considering the pore size distribution on the estimates MD fluxes. They state that the uncertainties in modeling and experimental design tend to overshadow the influence of pore size distribution in DCMD (but not in VMD). The inaccuracy in the predicted water vapor flux due to neglecting the pore size distribution is closely related to the width of the distribution. For DCMD, where molecular diffusion usually dominates, the authors report that the discrepancy is limited to ~3.5% for membranes with a mean pore size of at least 100 nm and a geometric standard deviation of the pore size of 1.2.

### 3.3. Membrane Porosity and Pore Tortuosity

Membrane porosity *ε* is defined as the ratio of the volume of pores to the total volume of the membrane. In general, the larger the porosity, the larger the permeate flux across the membrane and the lower the conductive heat dissipation. In Membrane Distillation, *ε* ranges between 30 and 85% [60]. The membrane porosity *ε* can be determined, as suggested by Smolder and Franken [68], from density measurements using the following equation [68,69]:

(45) $$\varepsilon =1-\frac{\rho_{m}}{\rho_{pol}}$$

in which *ρ_m_* and *ρ_pol_* are the densities of the membrane and polymeric material, respectively, and the mass of the filling gas is neglected. More recently, Fan et al. [64] estimated the porosity of PVDF membranes by means of gravimetric measurements, using the following equation:

(46) $$\varepsilon =1-\frac{w/\rho_{pol}}{A\;\delta }$$

where *w*, *A* and *δ* are the mass, membrane area and thickness of the membrane sample, respectively, and *ρ_pol_* is the density of the bulk polymer. Note that Equation (46) can be traced back to the more general Equation (45). Zou et al. [70] measured the porosity *ε* of a PVDF hollow fiber membrane using a kerosene immersion method, in which *ε* was evaluated by the following equation:

(47) $$\varepsilon =\frac{4\left(w_{2}-w_{1}\right)}{\pi \left(OD^{2}-ID^{2}\right)L\rho_{k}}$$

in which *w*_2_ and *w*_1_ are the masses of the membrane after and before the immersion in kerosene, respectively; *OD* and *ID* are the outer and inner diameters of the hollow fibers, respectively; *L* is the length of the hollow fibers; and *ρ_k_* is the density of kerosene.

Pore tortuosity *τ* is defined as the ratio of pore length *l* to membrane thickness *δ*. As remarked by Field et al. [71], it can play an important role in determining the membrane permeance to water vapor (see following Section 4). In general, the larger the pore tortuosity, the lower the permeate flux across the membrane. Pore tortuosity can be experimentally measured using gas permeation methods [72], but only under a considerable amount of assumptions, and the results often depend on the specific experimental technique adopted. Often, *τ* is simply assumed to be equal to 2. Khayet and Matsuura [28] observe that “*…in various theoretical models the tortuosity factor was used as an adjusting parameter to obtain better agreement between the simulated and the experimental permeate fluxes. In fact, it is difficult to measure the pore tortuosity*.”

Chang et al. [51], Khalifa et al. [40] and Yadav et al. [39] assume *τ* to be the inverse of the porosity *ε*:

(48) $$\tau =\frac{1}{\varepsilon }$$

Other authors, including Srisurichan et al. [73], Alkhudhiri et al. [8], Lai and Zhang [47] and Kariman et al. [48], correlate the tortuosity *τ* to the porosity *ε* using the correlation suggested by Macki and Meares [74]:

(49) $$\tau =\frac{{(2-\varepsilon )}^{2}}{\varepsilon }$$

For example, for the case of Liqui-Cel^®^ hollow fiber membrane modules, Bouguecha et al. [44] report a tortuosity *τ* = 2.6 with a porosity *ε* = 0.4. Calculating the pore tortuosity via Equation (49) would yield the broadly different value *τ* = 6.4, while computing *τ* as the inverse of porosity, Equation (48), yields the more correct value of 2.5.

### 3.4. Liquid Entry Pressure

**Figure 12:**
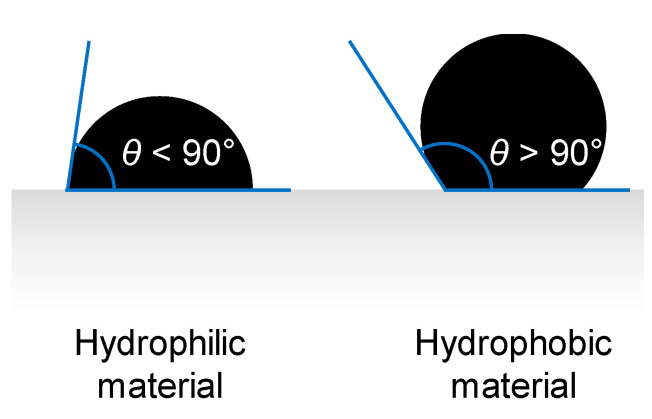
Schematic representation of the contact angle between a droplet of liquid and the membrane surface made by a hydrophilic or a hydrophobic material.

Liquid Entry Pressure (*LEP*) is a key membrane characteristic in MD. It is the threshold transmembrane pressure *p_f_* − *p_p_* (in which the subscripts “*f*” and “*p*” are for feed and permeate, respectively), above which the feed liquid begins to pass across the pores of the hydrophobic membrane. It depends on the membrane hydrophobicity and pore size. To calculate the *LEP* value, Alkhudhiri et al. [8], Zhang et al. [53], Ciofalo et al. [75] and many other authors report an expression proposed by Franken et al. [76] and derived from the Young–Laplace equation:

(50) $$LEP=-\frac{2B\Gamma \cos\theta }{r_{\max}}$$

in which *B* is a geometric pore coefficient (*B* = 1 for cylindrical pores), *Γ* is the liquid surface tension, *θ* is the contact angle between the solution and the membrane surface (see Figure 12) and *r_max_* is the largest pore radius. Lopez et al. [77] provide for *LEP* an equation identical to Equation (50) apart from the nomenclature.

For the contact between a single water droplet and a polymeric surface, for PTFE, Alkhudhiri et al. [8] report a value of *θ* in the range 108–115°; for PVDF, both Curcio and Drioli [10] and Tomaszewska [78] suggest a value of *θ* = 107°; for PP, Curcio and Drioli [10] give *θ* = 120°. In regard to the surface tension *Γ*, as reported by Sharkawy et al. [33], data for a saline solution are difficult to obtain and are scarce and rather scattered in the literature above a temperature of 35–40 °C. Zhang et al. [53], later followed also by Alkhudhiri et al. [8], express the influence of the molarity *M* on the surface tension as follows:

(51) $$\Gamma =\Gamma_{0}+\frac{\Delta \Gamma }{\Delta M}M$$

in which *M* is in mol/L, *Γ*_0_ is the surface tension of pure water (72 mN/m at 25 °C) and the value of Δ*Γ*/Δ*M* = 1.467 (mN/m)/(mol/L) was experimentally measured for NaCl solution by Sghaier et al. [79]. The influence of the dissolved salt is tiny up to seawater concentration; e.g., for *M* = 0.6 mol/L, one has *Γ* = 72.88 mN/m.

Rácz et al. [80] reported experimental *LEP* values for a variety of commercially available membranes including PTFE and PVDF flat-sheet and PP hollow fiber configurations, with mean pore diameters between 0.2 and 1.0 μm. Measured values of *LEP* ranged between 0.48 and 4.6 bar, with no correlation with material and geometry and only a loose correlation with the mean pore size.

## 4. Transmembrane Mass Transfer

**Figure 13:**
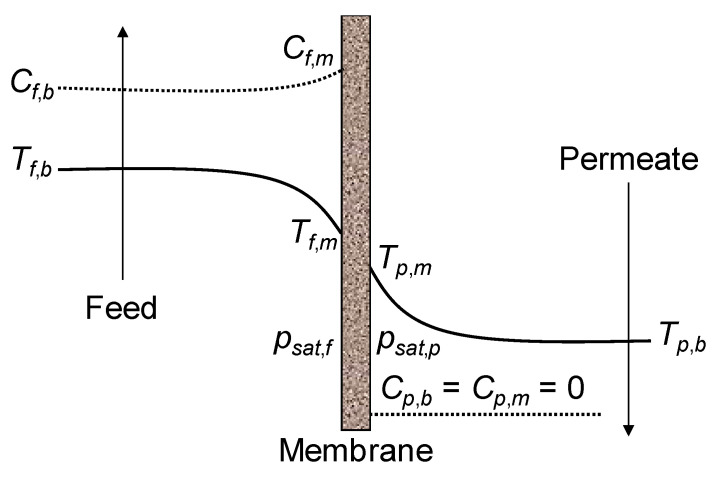
Schematic representation of the profiles of temperature (solid line) and concentration (broken line) on the two sides of the membrane.

With reference to Figure 13 below, the molar water vapor flux *J_vap_* through the membrane, expressed in mol m^−2^ s^−1^, can be modeled as follows:

(52) $$J_{vap}=C_{m}\left(p_{sat,f}\;-\;p_{sat,p}\right)$$

where *p_sat,f_* and *p_sat,p_* are the vapor saturation pressures corresponding to the temperatures and salt concentrations on the feed–membrane and permeate–membrane interfaces, respectively, expressed in Pa, and *C_m_* is the membrane permeance, expressed, consistently, in mol m^−2^ s^−1^ Pa^−1^.

Permeance is closely related to the mean free path of the water vapor molecules. The mean free path *λ* (expressed in m) of a molecule in a one-component gas, as given by Khayet and Matsuura [28], Alkhudhiri et al. [8] and several other authors, is as follows:

(53) $$\lambda =\frac{K_{B}T}{\sqrt{2}\pi p\sigma^{2}}\;$$

where *K_B_* is the Boltzmann constant (1.380649 × 10^−23^ J K^−1^), *T* is the absolute temperature, *p* is the mean pressure in a pore (in Pa) and *σ* is the collision diameter of the molecule. Cussler [81], Mills [82] and Momeni et al. [41] report *σ_vap_* = 2.641·10^−10^ m and *σ_air_* = 3.711·10^−10^ m. A slightly lower value is reported by Alkhudhiri et al. [8] for *σ_air_* (3.66·10^−10^ m). For example, for *p* = 1 bar (10^5^ Pa) and *T* = 323.15 K (50 °C), Equation (53) yields for *λ* a value of 1.44·10^−7^ m, or 0.144 μm, for water vapor and 0.73·10^−7^ m, or 0.073 μm, for air.

For a binary gas mixture (in particular, air–water vapor), several authors, including Phattaranawik and Jiraratanon [63], Khayet and Matsuura [28], Ghaleni et al. [83], Dong et al. [65], Momeni et al. [41] and Kariman et al. [48], use the following formula:

(54) $$\lambda_{g}=\frac{K_{B}T}{\pi p{\left(\frac{\sigma_{vap}+\sigma_{air}}{2}\right)}^{2}}\frac{1}{\sqrt{1+\frac{MW_{vap}}{MW_{air}}}}$$

For example, for a binary 50–50% mixture of air and water vapor under the same conditions as above (*p* = 1 bar, *T* = 323.15 K), Equation (54) yields *λ_g_* = 1.11·10^−7^ m, a value intermediate, as expected, between those computed for the two separate components.

**Figure 14:**
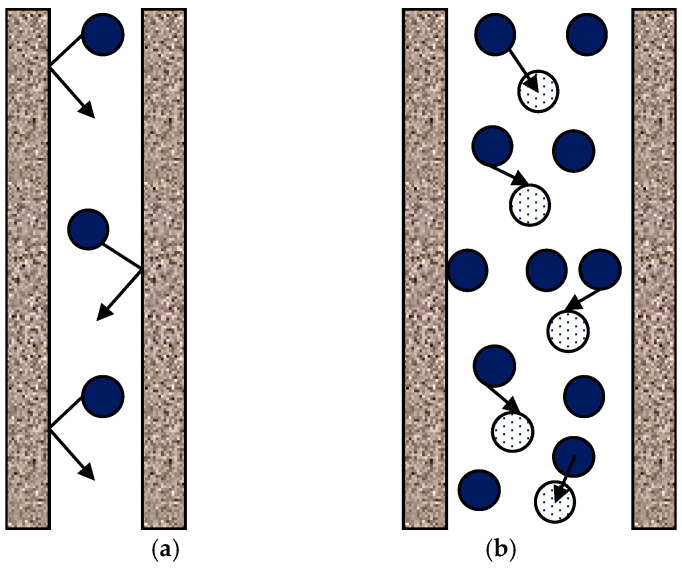
Transport mechanism through a pore of a membrane: (**a**) Knudsen regime and (**b**) diffusion regime.

The membrane permeance *C_m_* has different expressions depending on the value of the Knudsen number Kn = *λ*/*d_p_*, the ratio of the mean free path *λ* to the mean pore diameter *d_p_*. Two extreme cases are sketched in Figure 14.

Under conditions representative of MD, the mean free path of the water vapor molecules in a pore is of the order of 0.1 μm. As remarked by Dong et al. [65], since pore sizes typically range between 0.1 and 1 μm, the Knudsen number typically ranges between 0.1 and 1, thus falling in the intermediate, or transitional, region between the two above limiting cases. Similar conclusions are reached by Momeni et al. [41].

Thus, three cases are considered in the literature. They will be discussed in Section 4.1 through Section 4.3 here below.

### 4.1. Case I (Small Pores, Knudsen Regime): d_p_ < λ or Kn > 1

According to most authors, e.g., Hitsov et al. [32] and Dong et al. [65], in this case (Knudsen regime), molecule–pore wall collisions dominate with respect to molecule–molecule collisions. Some authors, e.g., Khayet and Matsuura [28], apply a more stringent criterion considering the Knudsen regime to be dominant only if Kn > 10, i.e., *d_p_*/*λ* < 0.1. Phattaranawik et al. [55] express the membrane permeance *C_m_*, defined by Equation (52) but written as *C_m_^Kn^* for “Knudsen”, in terms of a diffusivity *D^Kn^*:

(55) $$C_{m}^{Kn}=\frac{D^{Kn}}{R_{g}T\delta }$$

In its turn, *D^Kn^* (m^2^ s^−1^) is expressed as follows:

(56) $$D^{Kn}=\frac{4}{3}\frac{\varepsilon d_{p}}{\tau }\sqrt{\frac{R_{g}T}{2\pi MW_{vap}}}$$

where *ε* is the membrane porosity, *τ* is the pore tortuosity, *T* is the absolute temperature, *R_g_* is the universal gas constant (8.314 J mol^−1^·K^−1^) and *MW_vap_* is the molecular weight of water (18·10^−3^ kg/mol). In regard to *d_p_*, in [55], it is defined as the mean diameter of those pores that lie in the Knudsen portion of the pore size distribution (i.e., whose diameter is <*λ*); however, for simplicity, it can be identified with the mean pore diameter (implicitly assuming that all pores fall in the Knudsen regime).

From Equations (55) and (56) it follows that the membrane permeance *C_m_^Kn^*, expressed in mol m^−2^ s^−1^ Pa^−1^, can be explicitly written as follows:

(57) $$C_{m}^{Kn}=\frac{1}{3}\;\;\frac{\varepsilon d_{p}}{\tau \delta }\sqrt{\frac{8}{\pi R_{g}T\;MW_{vap}}}$$

Dong et al. [65], in their Equation (11.a), Olatunji and Camacho [20], in their Equation (36), Chang et al. [51], in their Equation (2), and Yadav et al. [39], in their Equation (6a), all give expressions for the membrane permeance in the Knudsen regime in terms of mass flux (i.e., in kg m^−2^ s^−1^ Pa^−1^), which, once converted into molar permeance (i.e., divided by *MW_vap_*) and allowing for changes in notation, become identical to Equation (57). Khayet and Matsuura [28] give an expression, their Equation (10.17), for *B_m_^Kn^* (defined as the molar permeance in the Knudsen regime *per single pore*); this must be multiplied by the number of pores per unit area, equal to 4*ε*/(*π d_p_*^2^), to obtain the molar permeance per unit membrane area, *C_m_^Kn^*. The result, allowing for changes in notation, becomes again identical to Equation (57) above.

### 4.2. Case II (Large Pores, Diffusion Regime): d_p_ > 100λ or Kn < 0.01

In this case (molecular diffusion regime), molecule–molecule collisions dominate with respect to molecule–pore wall collisions, and water vapor transport occurs through the concentration-driven diffusion of vapor molecules through the filling gas (air + water vapor). Phattaranawik and Jiraratananon [63], assuming the log-normal distribution of pore diameters in Equation (44), observe that, for a mean free path *λ* ≈ 0.11 μm, this case can typically be ignored because it interests only a small fraction of the pores (those with *d_p_* > 11 μm). However, Phattaranawik et al. [55] presented a detailed analysis of this case. By considering, for generality, opposite fluxes of air and water vapor through the pores, they obtained, in the present notation, the following expression for *C_m_* (mol m^−2^ s^−1^ Pa^−1^), called *C_m_^D^* for “diffusion”:

(58) $$C_{m}^{D}=\;\;\frac{\varepsilon D_{g}}{\tau \delta R_{g}T}{\left[1-\left(\frac{p_{vap}}{p}\right)\left(1-\frac{J_{air}}{J_{vap}}\right)\right]}^{-1}$$

in which *J_vap_* and *J_air_* are the molar fluxes of water vapor and air, respectively, *p_vap_* is the partial pressure of water vapor (which, as stated in Section 3.1, can be identified with *p_sat_*(*T*)), *p* = *p_vap_* + *p_air_* is the total pressure of the gas filling the pores and *D_g_* is the gas diffusivity. Under the common assumption that air is trapped in the pores, one has *J_air_* = 0, and Equation (58) becomes the following:

(59) $$C_{m}^{D}=\frac{D_{g}}{R_{g}T}\;\;\frac{p}{p_{air}}\frac{\varepsilon }{\tau \delta }$$

Dong et al. [65], in their Equation (11.b), and Olatunji and Camacho [20], in their Equation (37), give expressions for the membrane permeance in the diffusive regime in terms of mass flux (i.e., in kg m^−2^ s^−1^ Pa^−1^), which, once converted into molar permeance (i.e., divided by *MW_vap_*) and allowing for changes in notation, become identical to Equation (59). Chang et al. [51] report a slightly different expression, see their Equation (2), in which *p_air_*/*p* is replaced by the equivalent quantity |*Y_m_*|_ln_ (log mean mole fraction of air). As for the previous case of the Knudsen regime, Khayet and Matsuura [28] give an expression, their Equation (10.18), for *B_m_^D^* (defined as the permeance in the diffusive regime *per single pore*); this must be multiplied by the number of pores per unit area, 4*ε*/(*π d_p_*^2^), to obtain the permeance per unit membrane area, *C_m_^D^*. The result, allowing for changes in notation, becomes again identical to Equation (59).

For the diffusivity *D_g_*, the Fuller equation for binary gas diffusion [81,84] can be used:

(60) $$D_{g}=1\cdot 10^{-7}\frac{T^{1.75}}{p}\frac{\sqrt{\frac{1}{MW_{vap}}+\frac{1}{MW_{air}}}}{{\left(V_{vap}^{1/3}+V_{air}^{1/3}\right)}^{2}}\;\;$$

in which *D_g_* is in m^2^/s, *T* is in K, *p* is in atm, *MW* is in g/mol and *V* is the so called “diffusion volume” in cm^3^/mol. Alkhudhiri et al. [8] report that *V* = 12.7 cm^3^/mol for water vapor and 20.1 cm^3^/mol for air; Poling et al. [85] report the similar values *V* = 13.1 cm^3^/mol for water vapor and 19.7 cm^3^/mol for air. For example, for air–water vapor at *p* = 10^5^ Pa (1 bar) and *T* = 300 K, Equation (60) yields *D_g_* = 2.54·10^−5^ m^2^/s.

As an alternative, the diffusivity of water vapor through the stagnant air in the pores can be estimated by the simpler formula reported by many authors, including Phattaranawik et al. [55], Khayet and Matsuura [28], Olatunji and Camacho [20], Dong et al. [65] and Kariman et al. [48]:

(61) $$D_{g}=1.895\cdot 10^{-5}\frac{T^{2.072}}{p}$$

in which *D_g_* is in m^2^/s, *T* is in K and *p* is in Pa. For *T* = 300 K and *p* = 1 atm., this yields *D_g_* = 2.57·10^−5^ m^2^/s, almost identical to the outcome of the more complex Equation (60).

Yet another correlation is proposed by Cussler [81] and Ansari et al. [42]:

(62) $$D_{g}=4.32\cdot 10^{-4}\frac{T^{1.5}}{p}$$

in which, as in Equation (61), *D_g_* is in m^2^/s, *T* is in K and *p* is in Pa. For example, for *p* = 10^5^ Pa (1 bar) and *T* = 300 K, Equation (62) yields *D_g_* = 2.24·10^−5^ m^2^/s, farther than Equation (61) from the more complete Equation (60).

### 4.3. Case III (Intermediate Pore Size, Transitional Regime): λ < d_p_ < 100λ or 0.01 ≤ Kn ≤ 1

**Figure 15:**
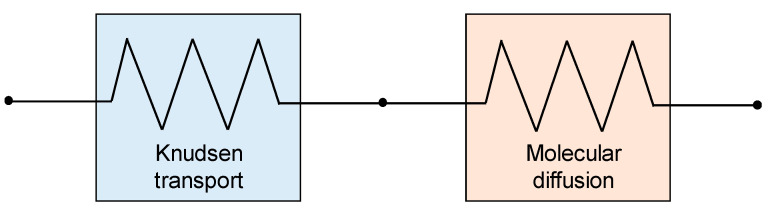
Transitional mass transfer regime with Knudsen transport and molecular diffusion in series.

In this case (transitional regime), both molecule–molecule collisions and molecule–pore wall collisions are important. Based on the Bosanquet theory for the overall self-diffusivity of a molecule under simultaneous Knudsen and molecular diffusion [86,87,88], and according to the majority of authors, e.g., Alkhudhiri et al. [8], Dong et al. [65], Chang et al. [51], Tsai et al. [89] and Kariman et al. [48], in this case, the resistances associated with molecular diffusion and Knudsen transport must be treated as resistances in series (Figure 15).

Consistently, the membrane molar permeance *C_m_* (now called *C_m_^C^* for “combined”) can be expressed as follows:

(63) $$\frac{1}{C_{m}^{C}}=\frac{1}{C_{m}^{Kn}}+\frac{1}{C_{m}^{D}}$$

Kuang et al. [46] report an explicit form of Equation (63) in which *C_m_^Kn^* is expressed using Equation (57), while *C_m_^D^* is expressed using Equation (59). The resulting expression of *C_m_^C^* (in mol m^−2^ s^−1^ Pa^−1^) is as follows:

(64) $$C_{m}^{C}=\frac{\varepsilon }{\tau \delta }{\left[\frac{3}{d_{p}}\sqrt{\frac{\pi R_{g}T\;MW_{vap}}{8}}+\frac{p_{air}}{p}\frac{R_{g}T}{D_{g}}\right]}^{-1}$$

Note that *C_m_^C^* depends on several quantities for which a more or less severe uncertainty exists. In particular, it is inversely proportional to the pore tortuosity *τ*, a quantity for which different formulae yield broadly different results (see Section 3.3). *C_m_^C^* is also directly proportional to the membrane porosity, which is also affected, albeit to a lesser extent, by uncertainty. Also, the choice of a single pore diameter *d_p_* is an oversimplification with respect to the actual pore size distribution (see remarks at the end of Section 3.2). Finally, also for the diffusivity *D_g_* and the ratio *p_air_*/*p*, different values are provided according to the model used.

Equation (64) must be multiplied by *MW_vap_* (in kg/mol) if one wishes to express *C_m_^C^* as *mass* permeance, i.e., as mass flux per unit pressure difference (in units of kg m^−2^ s^−1^ Pa^−1^), as, for example, in Olatunji and Camacho [20]. Similarly, Equation (64) has to be multiplied by the molar volume of liquid water *MW_w_*/*ρ_w_* (in m^3^/mol) if one wishes to express *C_m_^C^* as *volume* permeance, i.e., as liquid volume flux per unit pressure difference (i.e., in m s^−1^ Pa^−1^ units), as, for example, in Bouguecha et al. [44], who report for LiquiCel^®^ hollow fiber membranes a volume permeance of 3.27·10^−10^ m s^−1^ Pa^−1^.

### 4.4. Contribution of Poiseuille Flow

As a further transport mechanism, Poiseuille flow might be considered, see, e.g., Khayet and Matsuura [28]. However, this mechanism is in contradiction with the assumption that, at least in DCMD, air is trapped in the membrane pores; it is expected to be important only if this assumption is removed, and, even so, only in the presence of a significant transmembrane total pressure difference and very large pores, otherwise being negligible. As remarked by Field et al. [71], “*it is necessary to emphasize that the presence of Knudsen diffusion necessarily excludes the presence of Poiseuille flow*”. Also, according to Dong et al. [65], as seen in their Table 1, in the presence of a gas mixture (air–water vapor) in the pores with appreciable transmembrane partial vapor pressure difference but negligible total pressure difference (which are the conditions typical of DCMD), the only mass transport mechanisms to be considered are Knudsen transport for Kn > 1, molecular diffusion for Kn < 0.01, and a combination of these in the intermediate (and most common) range.

**Figure 16:**
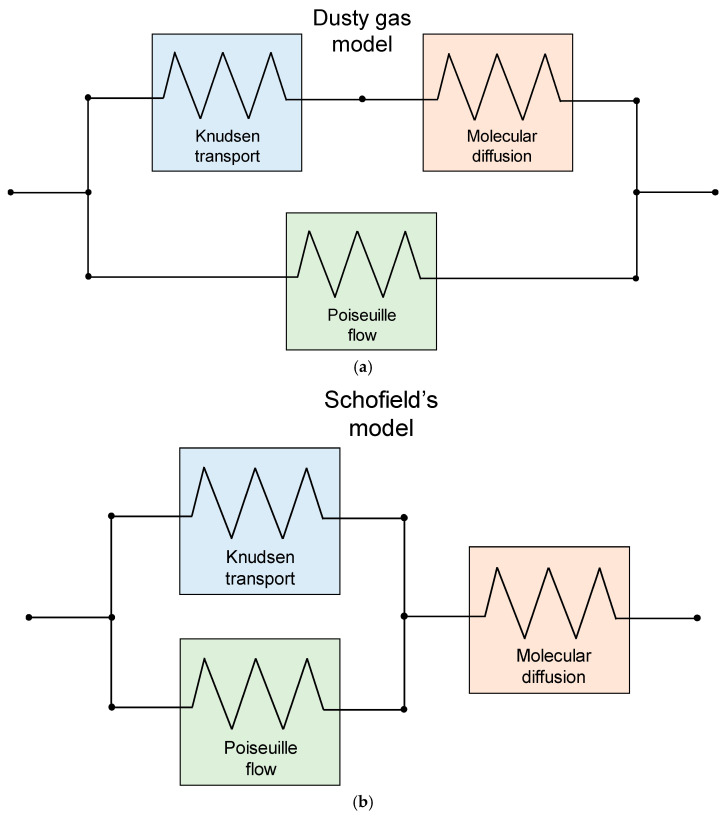
“Dusty gas” (**a**) and Schofield’s (**b**) models for mass transfer by Knudsen transport, molecular diffusion and Poiseuille flow.

However, only for the sake of completeness, we mention here that, in order to account for the simultaneous presence of Knudsen transport, molecular diffusion and Poiseuille flow, different models have been proposed, the most popular being the “Dusty gas” model (Figure 16a) and Schofield’s model [90] (Figure 16b). A detailed description of the Dusty gas model is given by Khayet and Matsuura [28], see their Equations (10.28) through (10.32). Expressions for the mass fluxes, as derived by both the above models, are given, among others, by Dong et al. [65]; see their Equations (9) and (10).

### 4.5. Experimental Validation of Permeance Models

Doubtlessly, the correlations which represent the core of this review and of the entire process of modeling Membrane Distillation are the expressions of the membrane permeance *C_m_* discussed in this section, because they directly affect the assessment of the process yield. Unfortunately, they are very difficult to validate experimentally: based on the definition of *C_m_*, validation would require measurements of the local vapor flux *J_vap_* and of local temperatures (and, for the feed side, also concentration) at the feed–membrane and permeate–membrane interfaces, from which local values of the vapor saturation pressure *p_sat_* on the two interfaces could be reliably calculated. These quantities are hardly accessible, which explains why relevant data are almost non-existent in the literature. In most cases, the available experimental information regards overall water vapor fluxes in a whole module and bulk values of temperatures and concentrations. Therefore, any experimental validation of a model for the membrane permeance can only be indirect and is affected by all the uncertainties associated with the large-scale spatial variation in all quantities within a module and, what is even more relevant, with temperature and concentration polarization (i.e., boundary layer resistance to the transport of heat and mass). Usually, authors presenting experimental values of *C_m_* or using this quantity within predictive models either neglect the boundary layer temperature drops [44] or treat *C_m_* as an adjustable parameter to be tuned on the basis of the overall agreement with experiments [91,92].

## 5. Transmembrane Heat Transfer

**Figure 17:**
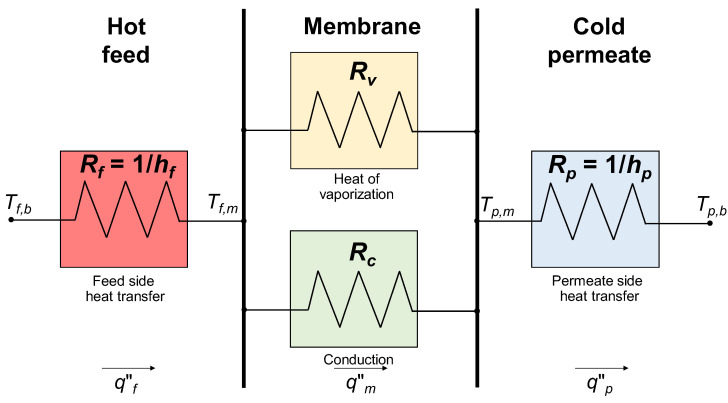
Heat transfer resistance model in DCMD.

In regard to the transmembrane heat transfer process, most authors, e.g., Khayet and Matsuura [28], Alkhudhiri et al. [8], Olatunji and Camacho [20], Dong et al. [65] and Cancilla et al. [93], represent it as the flow of heat through a combination of resistances, as shown in Figure 17. Three main steps are involved:

(a) Heat transfer from the feed bulk to the feed–membrane interface;
(b) Heat transfer from the feed–membrane interface to the membrane–permeate interface;
(c) Heat transfer from the membrane–permeate interface to the permeate bulk.

The following Equations (65)–(68) express the above heat transfer resistance model in formula form.

(a) The quantity *q*″*_f_*, expressed in W/m^2^, represents the heat flux from the bulk of the feed side channel to the feed–membrane interface:

(65) $$q_{f}^{\prime \prime }=h_{f}\left(T_{f,b}-T_{f,m}\right)$$

in which *h_f_* is the heat transfer coefficient of the feed compartment and *T_f_*_,*b*_ and *T_f_*_,*m*_ are the temperatures at the bulk feed and feed–membrane interface, respectively. The inverse of the heat transfer coefficient *h_f_* represents the thermal resistance *R_f_* associated with the hot feed side compartment in Figure 17.

(b) The quantity *q*″*_m_*, expressed in W/m^2^, represents the heat flux through the membrane. It is given by the parallel of two mechanisms:

- Heat flux due to the conduction across the polymeric membrane material and the gas-filled pores, which is denoted here as *q*″*_c_*;
- Heat flux due to the latent heat associated with the water vapor, which is denoted here as *q*″*_v_*.

The resulting total heat flux through the membrane wall can be written as follows:

(66) $$q_{m}^{\prime \prime }=q_{c}^{\prime \prime }+q_{v}^{\prime \prime }=\frac{k_{m}}{\delta }\left(T_{f,m}-T_{p,m}\right)+J_{vap}H_{fg}$$

where *k_m_* is the membrane thermal conductivity, *δ* is the membrane thickness, *T_p_*_,*m*_ is the membrane–permeate interface temperature, *J_vap_* is the water vapor flux (Equation (52)) and *H_fg_* is the latent heat of vaporization. The ratio *k_m_*/*δ* in Equation (66) represents the inverse of the thermal resistance *R_c_* associated with the conductive heat transfer through the membrane in Figure 17. The product *J_vap_H_fg_* is inversely proportional to the thermal resistance *R_v_* associated with the latent heat of vaporization in Figure 17. Note that all uncertainties on the membrane thermal conductivity directly affect both the heat flux and the ratio of sensible to latent heat fluxes.

According to Khayet and Matsuura [28], between 50% and 80% of energy is consumed as latent heat for water vapor production (*q*″*_v_*), while the remainder is lost by thermal conduction (*q*″*_c_*). In fact, the heat used effectively in DCMD is the latent heat of vaporization associated with the mass flux, whilst the heat transferred by conduction across the membrane is considered as heat loss. This heat loss becomes less significant at higher operating feed temperatures, and the thermal efficiency, *η*, of a DCMD process defined by the following Equation (67) becomes higher.

(67) $$\eta \left(\%\right)=\frac{q_{v}^{\prime \prime }}{q_{v}^{\prime \prime }+q_{c}^{\prime \prime }}\times 100$$

All temperature-dependent quantities explicitly or implicitly involved in Equation (66) can be evaluated at the membrane arithmetic mean temperature 〈*T_m_*〉 = (*T_f,m_* + *T_p,m_*)/2. As an alternative, following Phattaranawik and Jiraratananon [63], the geometric mean membrane temperature (*T_f,m_·T_p,m_*)^½^ can be used.

(c) The quantity *q*″*_p_*, expressed in W/m^2^, represents the heat flux from the membrane–permeate interface to the bulk of the permeate compartment:

(68) $$q_{p}^{\prime \prime }=h_{p}\left(T_{p,m}-T_{p,b}\right)$$

in which *h_p_* is the heat transfer coefficient of the permeate compartment and *T_p_*_,*b*_ is the temperature at the bulk permeate. The inverse of the heat transfer coefficient *h_p_* represents the thermal resistance *R_p_* associated with the cold permeate side compartment in Figure 17. Under steady state conditions,

(69) $$q_{f}^{\prime \prime }=q_{m}^{\prime \prime }=q_{p}^{\prime \prime }=q^{\prime \prime }$$

Note that, in modules adopting the hollow fiber membrane geometry, the above equations need to be adjusted to reflect the differences in membrane diameter and surface area between the inner (lumen side) and outer (shell side) surfaces.

The mass flux *J_vap_* can be expressed (and, if necessary, converted to kg m^−2^ s^−1^) by Equation (52), using the expressions for the vapor saturation pressure and for *C_m_* discussed in the previous Section 2 and Section 4, respectively. Due to the nonlinear dependence of the vapor saturation pressure on the temperature, the resulting set of equations is nonlinear and can only be solved by some iterative procedure. As an alternative, for a low transmembrane bulk temperature difference, many authors [8,28,51,94] linearize the dependence of *p_sat_* on *T* by writing the water vapor flux *J_vap_* in Equation (52) as follows:

(70) $$J_{vap}\approx C_{m}\frac{dp_{sat}}{dT}\left(T_{f,m}-T_{p,m}\right)$$

where the derivative of *p_sat_* with respect to *T* can be expressed by the Clausius–Clapeyron equation as follows:

(71) $$\frac{dp_{sat}}{dT}=\frac{H_{fg}}{R_{g}T^{2}}p_{sat}\left(T\right)$$

More complex expressions for *dp_sat_*/*dT*, suited for concentrated solutions, are proposed by Schofield et al. [54]. Linearizing *p_sat_* as in Equation (70) is only a crude approximation, especially if the temperature varies broadly, but leads to great simplifications, allowing the use of a global heat transfer coefficient incorporating both the latent heat associated with vapor transport and sensible heat associated with conduction. In this way, both terms *q*″*_c_* and *q*″*_v_* in Equation (66) become proportional to *T_f,m_* − *T_p,m_*. Thus, accounting for the vapor flux is equivalent to assuming for the membrane an *augmented* thermal conductivity *k_aug_*, expressed as follows:

(72) $$k_{aug}=k_{m}+C_{m}\delta {\left.\frac{dp_{sat}}{dT}\right|}_{\langle T_{m}\rangle }H_{fg}$$

### 5.1. Temperature Polarization Phenomena

Although the present review is focused on transmembrane heat and mass transfer in Membrane Distillation, a brief mention is appropriate of the heat transfer resistances exhibited by the boundary layers, which are recognized as a source of loss of DCMD efficiency. The temperature polarization coefficient (*TPC*) is generally used to quantify their relative importance with respect to the total heat transfer resistance. With reference to the overall temperature profile shown in Figure 13, *TPC* is defined as follows:

(73) $$TPC=\frac{T_{f,m}-T_{p,m}}{T_{f,b}-T_{p,b}}$$

and reflects the reduction in the driving force (i.e., in the temperature difference *T_f,m_* − *T_p,m_*, resulting in the vapor pressure difference *p_sat_*_,*f*_ − *p_sat_*_,*p*_), which has a negative influence on the DCMD process effectiveness. In the ideal case, *TPC* should be equal to unity, but usually it is significantly lower.

If the film heat transfer coefficients *h_f_* and *h_p_*, associated with the feed and the permeate boundary layers, which appear in Equations (65) and (68), are large, the temperatures at the membrane surfaces approach those of the bulk fluids, *TPC* approaches unity (i.e., the temperature polarization effect is negligible) and the DCMD process is controlled by the mass transfer resistance of the membrane. On the contrary, if the heat transfer coefficients are small, the difference between the temperatures at the membrane surfaces and those of the bulk fluids is large, and the transmembrane temperature difference is low. In this case (*TPC* approaching zero), the temperature polarization effect is very significant, and the DCMD process is controlled by the heat transfer resistances of the boundary layers. Generally, for satisfactory DCMD modules, *TPC* values range between 0.4 and 0.7. This means that between 30% and 60% of the applied temperature difference is spent in the thermal boundary layers.

Several methods have been adopted to minimize the heat transfer resistances of the boundary layers, thus increasing the film heat transfer coefficients. These include the use of spacers (for the spiral wound and plate-and-frame module geometries) or other mixing promoters, or simply the adoption of high feed and permeate flow rates. Notably, spacers promote fluid mixing, thereby enhancing the efficiency of convective heat transfer. Since the pivotal work of Schock and Miquel [95] in 1987, substantial research efforts have been devoted to elucidating the influence of spacer geometry on fluid dynamics and heat or mass transport within membrane systems affected by polarization phenomena. During the years, conventional woven and overlapped spacer designs have been systematically investigated, both experimentally and numerically, to assess their effects on friction as well as on heat and/or mass transfer coefficients [96,97,98,99].

More recently, several researchers have also studied unconventional sphere-type spacers using both CFD simulations [100,101,102] and experiments [103]. CFD has also been used to examine innovative designs, including 3D-printed geometries with hole–pillar arrangements [104], arch-type [105], airfoil-shaped filaments [106] and twisted [107] geometries, and, very recently, pillar–filament configurations [108]. These investigations consistently demonstrate that such advanced designs can achieve superior performance with respect to conventional and commercially available spacers. The body of literature on spacer-filled channels and their effects on polarization phenomena is vast. A comprehensive review of these contributions lies beyond the scope of the present work; readers are referred to dedicated review articles [23,109,110] that focus specifically on this topic.

### 5.2. Concentration Polarization Phenomena

By analogy to the treatment of temperature polarization and with reference to the overall concentration profile shown in Figure 13, a concentration polarization coefficient (*CPC*) can be defined as follows:

(74) $$CPC=\frac{C_{f,m}}{C_{f,b}}$$

where *C_f,m_* and *C_f,b_* are the salt concentrations at the feed–membrane interface and at the bulk feed, respectively. Since the membrane retention is ideally unity, the concentration at the permeate–membrane interface (*C_p,m_*) is equal to that in the permeate bulk (*C_p,b_*), and both are zero. For this reason, these two concentrations are omitted in Equation (74).

As anticipated in the Introduction, in DCMD, the concentration boundary layers make a smaller contribution to the overall mass transfer resistance, compared with temperature polarization [28]. The reason is that the vapor saturation pressure is strongly dependent on temperature but only weakly dependent on concentration, as shown in Section 2.5. In the MD process, the use of moderate flow rates and high heat transfer coefficients can make the influence of concentration polarization negligible. Khayet et al. [31], using a microporous hydrophobic membrane and a semi-empirical method, reported that concentration polarization was insignificant in DCMD across a range of flow rates and temperatures on both sides of the membrane. The negligible influence of *CPC* relative to *TPC* has also been demonstrated for other membranes in numerous studies [26,28,29,30].

## 6. Conclusions

Direct Contact Membrane Distillation (DCMD) is a promising separation technology with a growing relevance in desalination, water treatment and resource recovery. However, despite decades of research, the modeling of heat and mass transfer phenomena across DCMD systems remains fragmented, with significant variability in the models, assumptions, equations and correlations used.

This review has systematically analyzed recent and classical models, highlighting inconsistencies, overlaps and gaps. As anticipated in the Introduction, this comprehensive appraisal reveals that, while numerous models exist, many can be reconciled by recognizing equivalent formulations, excluding non-justified or even non-physically based equations, and acknowledging a few prevalent errors in past studies.

In particular, assumptions which are shared by most of the literature on DCMD are the following:

- The membrane pores are filled with a binary mixture of water vapor (moving from feed to permeate) and still, trapped air;
- The mean free path of water (vapor) molecules in the pores can be evaluated by Equation (54);
- The diffusivity of water (vapor) molecules in the pores can be evaluated by Equation (60) (Fuller equation) in the molecular diffusion regime and by Equation (56) in the Knudsen regime. Also, the resulting expressions for the membrane permeance, Equations (57) and (58), are shared by almost all authors (apart from the choice of units and other details);
- The Liquid Entry Pressure (*LEP*) can be estimated as a function of the surface tension, the pore radius and the solution–membrane contact angle using Equation (50).

For other aspects, no general consensus exists in the literature:

- Accounting or not accounting for Poiseuille flow in transmembrane mass transfer; see, e.g., Khayet and Matsuura [28] and Ansari et al. [42] vs. Dong et al. [65] and Field et al. [71];
- Linearizing or not linearizing the dependence of the vapor saturation pressure on temperature in Equation (70) for the water vapor flux; see, e.g., Alkhudhiri et al. [8], Khayet and Matsuura [28] and Field et al. [71] vs. Momeni et al. [41], Olatunji and Camacho [20] and Hitsov et al. [32];
- Choosing the temperature at which to evaluate the membrane properties, i.e., the arithmetic mean of *T_f,m_* and *T_p,m_* (most authors) or their geometric mean (Phattaranawik and Jiraratananon [63]);
- Predicting the membrane thermal conductivity as a function of porosity and of the conductivities of the polymeric matrix and the filling gas (Equations (31)–(34));
- Predicting the pore tortuosity as a function of porosity (Equation (48) versus Equation (49)).

By providing a comprehensive and rigorous comparative assessment, we aim to establish a common basis for DCMD modeling: a set of recommended equations, parameter values and best practices that researchers and engineers can reliably use for predicting performance. Hopefully, this will not only help researchers to resolve discrepancies and reduce errors in future simulations but also make MD modeling more accessible to scientists and engineers outside the immediate MD community.

A sensitivity analysis of the influence of thermophysical and kinetic correlations on the predicted performance of DCMD modules would require a model of the whole Membrane Distillation process, including the effects of temperature and concentration polarization on overall heat and mass transfer coefficients and the spatial distribution of residence times. This kind of analysis is beyond the scope of the present paper, which focuses on local transmembrane heat and mass transport models, and will be the subject of future studies. In our opinion, small differences between alternative correlations, e.g., in the thermophysical properties of salt water, would have a minor influence on the prediction of water vapor and heat fluxes, whereas other options, e.g., in the estimate of quantities like the pore tortuosity or membrane thermal conductivity, may severely affect the overall process performance.

Possible extensions of the present work, aimed at supporting the scale-up and implementation of DCMD in industrial applications, should include the following:

- A comparison of the proposed models and correlations for transmembrane transport with both experimental results and ab initio molecular dynamics predictions and other advanced approaches;
- An integration of the above results within more complete and fully predictive computational models accounting for the fluid dynamic aspects of the process. This will also make it possible to conduct sensitivity analyses on the influence of different correlations and model options on practical quantities such as freshwater yield and thermal consumption;
- A combination of heat and mass transfer models with economic and environmental assessment tools to support the scale-up of DCMD at a semi-industrial scale level;
- An exploration of new module designs using computational fluid dynamics, which should be evaluated alongside economic considerations such as manufacturing, operating and maintenance costs;
- The use of 3D-printed or nano-engineered modules could offer unprecedented control over membrane properties and module design, allowing for the creation of complex architectures customized for specific applications.

## Figures and Tables

**Table 1:** Thermal conductivity of common hydrophobic commercial membrane materials at different values of the mean membrane temperature 〈*t_m_*〉. From data in Hitsov et al. [32].

Polymeric Material	Constants	*k_sm_* at 20 °C (W m^−1^ K^−1^)	*k_sm_* at 40 °C (W m^−1^ K^−1^	*k_sm_* at 60 °C (W m^−1^ K^−1^)	*k_sm_* at 80 °C (W m^−1^ K^−1^)	*k_sm_* at 100 °C (W m^−1^ K^−1^)
Polypropylene (PP)	α = 12.5	0.13	0.16	0.18	0.21	0.23
	β = −23.5					
Polyethersulfone (PES)	α = 4.17	0.14	0.15	0.15	0.16	0.17
	β = 1.45					
Polyvinylidene fluoride (PVDF)	α = 5.77	0.18	0.19	0.20	0.21	0.22
	β = 0.914					
Polytetrafluoroethylene (PTFE)	α = 5.77	0.26	0.27	0.28	0.29	0.30
	β = 8.914					

## Data Availability

The raw data supporting the conclusions of this article will be made available by the authors on request.

## Nomenclature

*A*	Membrane area (m^2^)
*A*_1_, *B*_1_	Coefficients in the dynamic viscosity correlation, Equation (11)
*A*_2_, *B*_2_, *C*_2_, *D*_2_	Coefficients in the specific heat capacity correlation, Equation (15)
*a_w_*	Water activity (-)
*B*	Geometric pore coefficient in Equation (50) (-)
*C*	Molar concentration, Equation (4) (mol/m^3^)
*C_m_*	Membrane permeance (kg m^−2^ h^−1^ Pa^−1^ or mol m^−2^ h^−1^ Pa^−1^)
*c_p_*	Specific heat capacity (J kg^−1^ K^−1^)
*CPC*	Concentration polarization coefficient, Equation (74) (-)
*D*	Diffusivity (m^2^/s)
*d_p_*	Pore diameter (m)
*h*	Heat transfer coefficient (W m^−2^ K^−1^)
*H_fg_*	Latent heat of vaporization (J kg^−1^)
*ID*	Inner fiber diameter of the fiber (m)
*J*	Mass / molar flux (kg m^−2^ h^−1^ / mol m^−2^ h^−1^)
*k*	Thermal conductivity (W m^−1^ K^−1^)
*K_B_*	Boltzmann’s constant (J K^−1^)
Kn	Knudsen number (-)
*L*	Length of the hollow fiber membrane (m)
*l*	Pore length of the membrane (m)
*LEP*	Liquid Entry Pressure, Equation (50) (Pa)
*M*	Molarity, Equation (3) (mol/L)
*m*	Molality, Equation (2) (mol/kg)
*MW*	Molecular weight (g/mol)
*OD*	Outer fiber diameter of the fiber (m)
*p*	Pressure (Pa)
*p_sat_*	Vapor saturation pressure (Pa or atm)
*q*″	Heat flux (W/m^2^)
*R*	Heat transfer resistance (m^2^ K W^−1^)
*R_g_*	Universal gas constant (J mol^−1^ K^−1^)
*r_max_*	Largest pore radius (m)
*S*	Salinity (or mass fraction) (kg/kg)
*SD_log_*	Standard deviation of lognormal function (-)
*T*	Absolute temperature (K)
*t*	Temperature (°C)
*TPC*	Temperature polarization coefficient, Equation (73) (-)
*V*	Diffusion volume (cm^3^/mol)
*w*	Membrane mass (kg)
*x*	Molar fraction, Equation (1) (mol/mol)
Greek symbols	
*α*, *β*	Coefficients in Equation (42)
*Γ*	Surface tension (mN/m)
*γ_ω_*	Activity coefficient (-)
*δ*	Membrane thickness (m)
*ε*	Porosity of the membrane (-)
*ζ*	Coefficient in Equations (33) and (34) (-)
*η*	Thermal efficiency (-)
*θ*	Contact angle (degree)
*λ*	Mean free path (m)
*μ*	Dynamic viscosity (Pa s)
*ρ*	Density (kg/m^3^)
*σ*	Collision diameter of the molecule (m)
*τ*	Pore tortuosity of the membrane (-)
Subscripts	
*air*	Air
*aug*	Augmented
*b*	Bulk
*c*	Conduction
*f*	Feed
*g*	Gas
*k*	Kerosene
*m*	Membrane
*max*	Maximum
*p*	Permeate
*pol*	Polymeric material
*s*	Salt
*sat*	Saturation
*sm*	Solid matrix
*v*	Vaporization
*vap*	Water vapor
*w*	Water
0	Referred to pure water
1	Referred to before the immersion in kerosene
2	Referred to before the immersion in kerosene
Superscripts	
*C*	Referred to the transitional (combined between diffusion and Knudsen) region
*D*	Referred to diffusion region
*Kn*	Referred to Knudsen region
Averages	
〈 〉	Mean value

## Abbreviations

The following abbreviations are used in this manuscript:

CFD	Computational Fluid Dynamics
CPC	Concentration Polarization Coefficient
DCMD	Direct Contact Membrane Distillation
LEP	Liquid Entry Pressure
MD	Membrane Distillation
PES	Polyethersulfone
PP	Polypropylene
PTFE	Polytetrafluoro-ethylene
PVDF	Polyvinylidene fluoride
TPC	Temperature Polarization Coefficient
